# Lifelong aerobic exercise protects against inflammaging and cancer

**DOI:** 10.1371/journal.pone.0210863

**Published:** 2019-01-25

**Authors:** Mats I. Nilsson, Jacqueline M. Bourgeois, Joshua P. Nederveen, Marlon R. Leite, Bart P. Hettinga, Adam L. Bujak, Linda May, Ethan Lin, Michael Crozier, Daniel R. Rusiecki, Chris Moffatt, Paul Azzopardi, Jacob Young, Yifan Yang, Jenny Nguyen, Ethan Adler, Lucy Lan, Mark A. Tarnopolsky

**Affiliations:** 1 Department of Pathology and Molecular Medicine, McMaster University Medical Center (MUMC), Hamilton, Ontario, Canada; 2 Exerkine Corporation, McMaster University Medical Center (MUMC), Hamilton, Ontario, Canada; 3 Department of Pediatrics, McMaster University Medical Center (MUMC), Hamilton, Ontario, Canada; University of Rome La Sapienza, ITALY

## Abstract

Biological aging is associated with progressive damage accumulation, loss of organ reserves, and systemic inflammation ('inflammaging'), which predispose for a wide spectrum of chronic diseases, including several types of cancer. In contrast, aerobic exercise training (AET) reduces inflammation, lowers all-cause mortality, and enhances both health and lifespan. In this study, we examined the benefits of early-onset, lifelong AET on predictors of health, inflammation, and cancer incidence in a naturally aging mouse model (C57BL/J6). Lifelong, voluntary wheel-running (O-AET; 26-month-old) prevented age-related declines in aerobic fitness and motor coordination vs. age-matched, sedentary controls (O-SED). AET also provided partial protection against sarcopenia, dynapenia, testicular atrophy, and overall organ pathology, hence augmenting the ‘physiologic reserve’ of lifelong runners. Systemic inflammation, as evidenced by a chronic elevation in 17 of 18 pro- and anti-inflammatory cytokines and chemokines (P < 0.05 O-SED vs. 2-month-old Y-CON), was potently mitigated by lifelong AET (P < 0.05 O-AET vs. O-SED), including master regulators of the cytokine cascade and cancer progression (IL-1β, TNF-α, and IL-6). In addition, circulating SPARC, previously known to be upregulated in metabolic disease, was elevated in old, sedentary mice, but was normalized to young control levels in lifelong runners. Remarkably, malignant tumours were also completely absent in the O-AET group, whereas they were present in the brain (pituitary), liver, spleen, and intestines of sedentary mice. Collectively, our results indicate that early-onset, lifelong running dampens inflammaging, protects against multiple cancer types, and extends healthspan of naturally-aged mice.

## Introduction

In the aftermath of the sociopolitical, scientific, and medical advances of the 20^th^ century, global fertility and mortality rates have steadily declined and average life-expectancies risen [1–3]. Albeit a remarkable human achievement *per se*, population demographics are also shifting in favor of older adults (≥60 y), and this group is expected to increase from 800 million to 2 billion in the next four decades, representing 22% of the total world population by 2050 [4]. Age-related disorders currently account for ≈25% of the global burden of disease with the leading contributors being the chronic, non-communicable diseases (NCDs), such as cardiovascular disease (CVD), cancer, musculoskeletal disorders (MSD), and mental/neurological conditions [5]. Because population aging is expected to impose a formidable challenge in terms of escalating health-care costs, and NCDs are the principal preventable cause of death and disability, a greater emphasis must be put on preventive medicine in the 21^st^ century [4].

Sedentary living and physical inactivity are highly prevalent and strongly related to old age [6]. As accelerants of the biological aging process, they predispose for chronic disease and are estimated to underlie 3.2–5.3 million deaths per year globally (i.e. ≈ 9% of all-cause mortality), with associated costs exceeding $67.5 billion [7, 8]. In contrast, physical activity (PA) protects against CVDs, cancers, MSDs, metabolic syndrome, depression, anxiety, and cognitive/neurodegenerative disorders; collectively, reducing all-cause mortality risk by ≈30–40% [9–15]. Specifically, aerobic exercise training (AET) delays the onset of morbidity and enhances both health and lifespan [16]. Epidemiological evidence suggest that as little as 10 to 20 minutes of leisure PA per day is sufficient to extend life expectancy [17], while vigorous AET (e.g., running) provides additional survival benefits up to 3–5 times the recommended PA minimum (75–150 min/wk), with up to 10-fold higher training volumes generally considered safe and well-tolerated [9, 10, 12, 18]. Runners have ≈45–70% and ≈30–50% reduced risks of CVD- and cancer-related mortality, respectively, and live ≈3–10% (2–8 y) longer than non-runners, even after adjusting for potential confounders such as other types of PA [19, 20]. Epidemiological studies have consistently verified significant pro-longevity effects of AET, and an ongoing longitudinal trial suggests morbidity compression in lifelong runners [21].

The multi-systemic benefits of AET are well-documented in the discipline of oncology and exercise has been shown to be protective against breast, endometrial, colon and prostate cancers [21–27]. Numerous other malignancies, including liver, lung, kidney, bladder, rectal, brain, neck, esophageal adenocarcinoma, gastric cardia, myeloid leukemia, and myeloma, were recently added to this list of cancers favourably influenced by AET [15]. For example, walking (19–37 km/wk) and running (12–25 km/wk) may lower the risk of fatal brain cancers by > 40% [28]. Although the underlying mechanisms behind the oncoprotective benefits of regular exercise remain largely unknown, AET may mitigate several distinct stages in cancer progression, such as tumor initiation, growth, and metastasis [29–32].

Many cellular processes associated with aging predispose to oncogenic transformation, including mitochondrial dysfunction, oxidative stress, somatic mutation burden, cell senescence, and systemic inflammation [33–38]. Age-related chronic, low-grade inflammation (‘inflammaging’) is characterized by a two- to four-fold increase in circulating cytokines, chemokines, growth factors, and proteases, henceforth termed ‘gerokines’. Depending upon the biological context, gerokines can be broadly classified into pro-inflammatory (TNF-α, IL-1α/β, IL-6, IL-8, IFN-γ, VEGF etc.) and/or anti-inflammatory (IL-2, IL-4, IL-10, IL-13, TGF-β etc.), both of which are predicted to contribute to the aging secretome [39–41]. Several gerokines are capable of modulating all stages of oncogenesis, from tumor initiation and growth to cancer invasion and metastasis [33, 42], particularly TNF-α and IL-6. Virtually all solid malignancies exhibit extensive immune cell infiltration and contain high levels of pro-inflammatory cytokines, growth factors, and remodeling proteins, making them attractive targets for adjuvant cancer therapies, such as cytokine blockade and exercise interventions [32, 43, 44].

Strategies to rejuvenate pivotal systems that govern the basic survival needs of the cell, including energy production, growth, and quality control (repair, folding, recycling/degradation, and synthesis), may significantly extend health- and lifespan. Acute aerobic exercise is a stress stimulus characterized by pulsatile changes in intracellular danger signals (Ca^2+^, ROS, pH, and hypoxia), metabolic intermediates (NAD+/NADH and AMP/ATP), and circulating factors (termed ‘exerkines’), that coordinately stimulate mitochondrial biogenesis, antioxidant defense, cellular repair and recycling mechanisms, and immunity [45–50]. Over time, AET results in physiological adaptations into a more stress-resistant, homeostatic level [51, 52], which likely protects against systemic inflammation and cancer development.

In the current study, we report that lifelong, voluntary wheel-running with onset at sexual maturity alleviated senescence and inflammaging, including the key drivers of the cytokine cascade and tumor progression (TNF-α, IL-6, and IL-1β), and protected against several different types of cancer (e.g., brain, liver, spleen, and intestinal) in naturally-aged mice. Although lifelong AET provided a pleiotropic stimulus, which attenuated the biological aging process of multiple organ systems (for example, heart, skeletal muscle, and testicles) and improved overall health, the median lifespan was only modestly enhanced in the runners. These results are integrated and discussed within the framework of major biological aging hypotheses, namely the entropic, garbage catastrophe, and inflammaging/remodelling models by Hayflick et al., Terman, and Franceschi, respectively [39, 53, 54].

## Materials and methods

### Ethics

All methods and procedures described herein followed the guidelines published by the Canadian Council of Animal Care and were approved by McMaster University’s Animal Research and Ethics Board under the Animal Utilization Protocol 12-03-09.

### Animals and study design

Four-week-old C57BL/J6 mice were purchased from Jackson Laboratories (Bar Harbor, ME), matched according to sex and bodyweight, and assigned to either young baseline control (Y-CON; N = 40), old sedentary (O-SED; N = 32), or old lifelong aerobic exercise (O-AET; N = 36) conditions. Following a 3-week acclimation period, O-AET animals were independently housed in activity wheel chambers and engaged in lifelong voluntary wheel-running, while baseline and sedentary cohorts were kept in separate microisolator cages with standard environmental enrichment. All mice were maintained on a 12-h light/dark cycle in a temperature and humidity-controlled room with water and chow available ad libitum (Harlan Teklad 8640 22/5). Sexual maturity (56 d) and old age (806 d) were used as endpoints for young and old cohorts, respectively, with the latter representing the lower limit of the 95% confidence interval for the median lifespan of C57BL/J6 mice [55, 56]. One week prior to necropsies, animals underwent a health examination and a battery of functional tests over two days to determine aerobic fitness, muscular strength and endurance, and motor coordination. A sub-set of mice from each group was terminally bled at rest or post exercise for assessment of age- and exercise-induced circulatory factors (Y-CON; n = 7, Y-CON-EX; n = 8, O-SED; n = 8, O-SED-EX; n = 6, O-AET; n = 9, and O-AET-EX; n = 8). To ensure a robust exercise stimulus in the young cohort, additional C57BL/J6 mice were obtained and exercised separately from the main-study animals until complete exhaustion and assessed independently for the secretory response (Y-CON_2_; n = 5 and Y-CON-EX_2_; n = 5).

### Necropsies and histopathology

All experimental conditions were equally represented each day of sacrifice, and the necropsy order was balanced for time-point between groups. Complete, macroscopic post-mortems were performed by a veterinary pathologist blinded to the group allocations. Carcasses were first evaluated externally prior to internal examination and collection of major organs of the respiratory (lungs), cardiovascular (heart), digestive (liver, gallbladder, and intestine), lymphatic (spleen), endocrine (pancreas), urogenital (kidney, urinary bladder, and gonads), integumentary (skin), musculoskeletal (skeletal muscle), and central nervous (brain and spinal cord) systems. Any suspected tumor or mass was formalin-fixed, embedded in paraffin, sectioned, and stained with haematoxylin and eosin (H&E) for histological examination. The H&E-stained slides were then sent to an anatomical pathologist for blinded microscope analyses. The tumors, based on histology, were classified as malignant, benign, or unclassified.

### Voluntary running-wheel training

O-AET mice were singly housed in 35.3L x 23.5W x 20H cm chambers equipped with free-spinning exercise wheels (40 cm/revolution) and counters connected to an activity monitoring software (Lafayette Instruments, Model 80820). Age-related changes in activity patterns were similar to those previously observed in C57BL/J6 mice [57, 58], and daily running distances were significantly reduced from middle adulthood onwards (S1 Fig). In a similar fashion to recent published work [59, 60], sedentary animals were independently housed without running-wheels to limit the complexity of the environment, spontaneous physical activity, and escape behaviors (e.g., climbing). Assessment of non-wheel cage activity during light and dark cycles using the Comprehensive Lab Monitoring System indicated no differences between O-SED and O-AET, while old animals were significantly less active versus young (S1 Table).

### Functional testing and maximal run protocol

One week prior to sacrifice, a battery of *in vivo* functional tests was administered to predict health and morbidity status of the mice, including muscular strength (‘dynapenia 1’) and endurance (‘dynapenia 2’), motor coordination, and aerobic fitness. Our group has validated these outcomes extensively in various mouse models, and performance variables generally reflect organ dysfunction and total disease burden [61, 62]. In brief, forelimb maximal strength (N) was tested using a digital grip strength meter equipped with a pull bar and a force gauge (Columbus Instruments, Columbus, OH). Motor coordination and dynamic balance were assessed using a conventional RotaRod system (AccuScan Instruments, Inc., Columbus, OH), in which the speed (RPM) and time (s) at fall were recorded. Muscular endurance was evaluated by a four limb, inverted grid-hang test until failure (s) (Paw Grip Endurance, PaGE). Lastly, maximal aerobic capacity was predicted in all mice using an incremental running test, which consisted of a ramped exercise protocol on a motorized treadmill starting at 10 m/min and increasing 1 m/min until volitional exhaustion (e.g., sitting/lying on the electrical grid). A separate cohort of young mice was exercised until complete exhaustion, defined as the inability to run on the treadmill despite 10 sec of mechanical prodding, to provide a robust stimulus for exercise factor release. All other parameters remained the same between tests.

### Serum secretome analyses

Blood was drawn from the retro-orbital sinus, clotted for 1 hr at room temperature (RT), spun at 2,500 g for 15 min at 4°C, and serum was retained for assessment of age- and exercise-induced cytokines, chemokines, and myokines. Serum samples were immediately frozen in liquid N_2_ and stored at -80°C until shipped on dry ice to the Analytical Facility for Bioactive Molecules (The Hospital for Sick Children, Toronto, Canada). Pro- and anti-inflammatory gerokines (GM-CSF, IFN-γ, IL-1α, IL-1β, IL-5, IL-6, IL-7, IL-12, IL-17A, CXCL1/KC, CXCL5/LIX, CCL2/MCP-1, CXCL2/MIP-2, and TNF-α vs. L-2, IL-4, IL-10, and IL-13, respectively) were analyzed by a research technologist using EMD Millipore’s (Temecula, CA, USA) high-sensitivity 18-plex plate (MHSTCMAG-70K) and were readily detected in all serum samples. Myokines were assessed on a separate 12-plex plate (MMYOMAG-74K) and a majority were above the lower limit of quantitation (LOQ), including FGF-21, FSTL-1, FKN/CX3CL1, IL-15, MSTN/GDF8, OSTN, and SPARC. Preparation, storage, and analyses were standardized between experimental conditions. Each sample consisted of serum from one or two mice within the same group and samples were run in duplicate on each plate.

### Cumulative health index rank

Predictors of successful aging, including cancer incidence, skin abscesses and lesions, other organ pathology, aerobic capacity, muscle mass (sarcopenia), grip strength (dynapenia 1), muscular endurance (dynapenia 2), and motor coordination were individually ranked on a 5-grade scale as follows: 1 = -51% or lower vs. Y-CON, 2 = -26% to -50% vs. Y-CON, 3 = -1% to -25% vs. Y-CON, 4 = 0% to +25% vs. Y-CON, and 5 = ≥ +26% vs. Y-CON. A cumulative health index rank was obtained by averaging the individual ranks, with indexes 1 and 5 representing most decrepit (1–2 = pathological aging) and excellent health status (3–5 = healthy aging), respectively.

### Statistical analyses

#### Parametric data

All parametric data were tested for normality and homogeneity of variance by Shapiro-Wilk and Levene’s tests, respectively. Two balanced, three-way ANOVAs were used to determine the independent and interactive effects of age, AET, acute exercise, and gender on all parametric data, including functional, morphometric, and secretory outcomes. Specifically, the 2 x 2 x 2 ANOVA designs were: 1. AGE x ACUTE EXERCISE x GENDER (young and old mice) and, 2: AET x ACUTE EXERCISE x GENDER (old mice). If omnibus F-test(s) were significant, a Fisher’s LSD post hoc test was used to specify group differences. Independent T-tests were used to assess the exercise-induced secretory response in a sub-set of young animals. Statistical significance was set at P ≤ 0.05 for all parametric tests.

#### Non-parametric data

For organ and skin pathology, frequencies were coded as binary data (i.e. present vs. non-present), evaluated by either Chi-square or Fisher’s Exact tests, and graphed as % incidences for each cohort ([total frequency of pathology/number of necropsies]*100). Cumulative health index ranks were analyzed by Kruskal Wallis and Dunn’s multiple comparison tests. Significance was set at P ≤ 0.05 for all non-parametric tests.

#### Graphs, tables, and denotations

For conciseness, groups were collapsed across gender and acute exercise and presented as Y-CON, O-SED, and O-AET when appropriate, with the complete statistical designs available in table format in the supplementary data section. Statistical differences are denoted alphabetically (a, b, and/or c) or symbolically (*, †, and ‡) and if group means are significantly different they do not share the same letter(s) or symbol(s) (P > 0.05).

## Results and discussion

### Aging, organ deterioration, and disability

**Fig 1:**
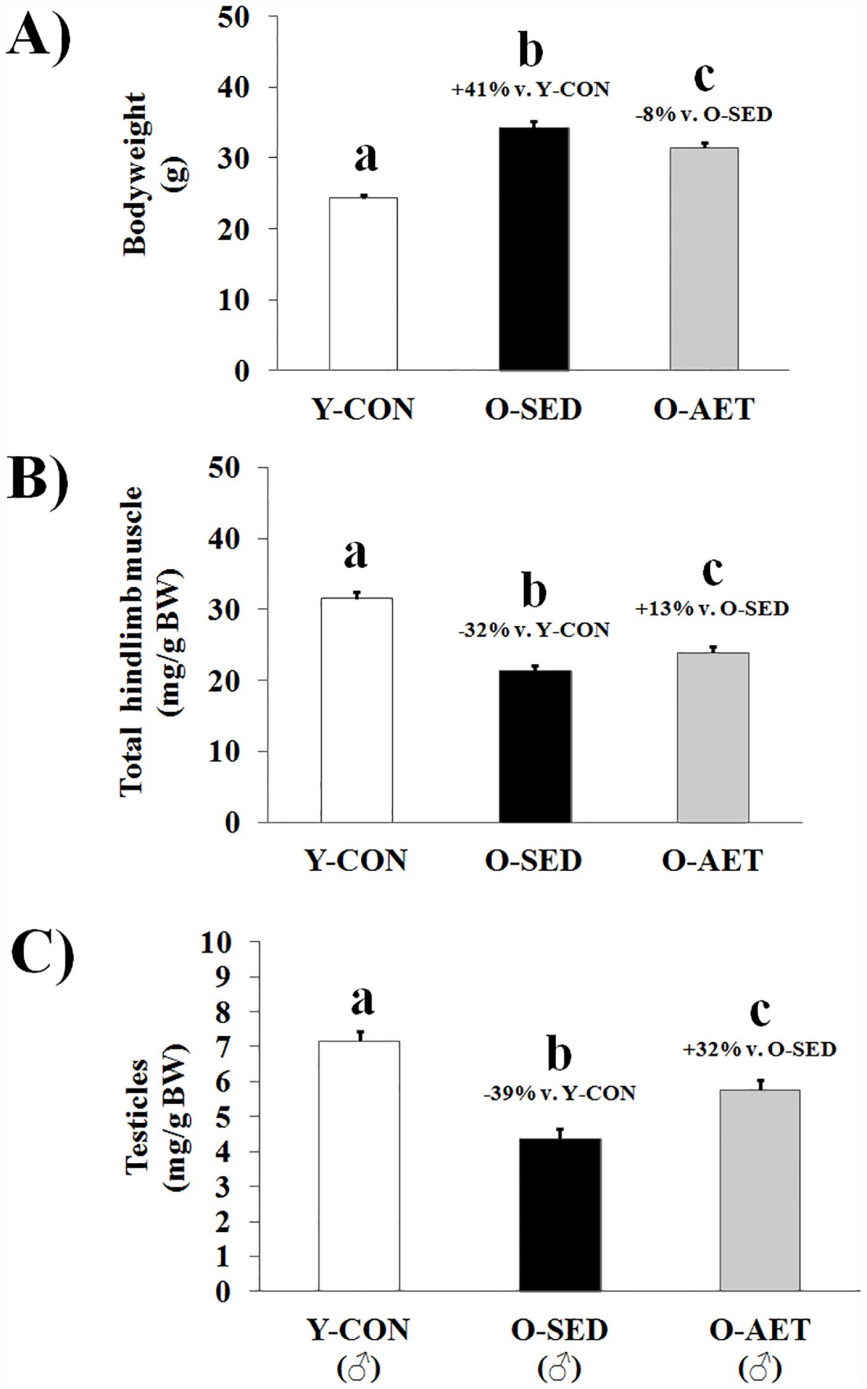
Lifelong aerobic exercise mitigates age-associated bodyweight gain, sarcopenia, and testicular atrophy. **A)** Bodyweight, **B)** hindlimb muscle mass, and **C)** testicle mass of young control (Y-CON), old sedentary (O-SED), and lifelong aerobically trained (O-AET) C57BL/J6 mice. Major thigh (quadriceps complex) and lower leg (anterior and posterior crural) muscles were summed for total hindlimb muscle mass. Complete data-sets are shown in S1 and S2 Tables. Group means (bars) that do not share the same alphabetical letter(s) are statistically different at P < 0.05.

**Fig 2:**
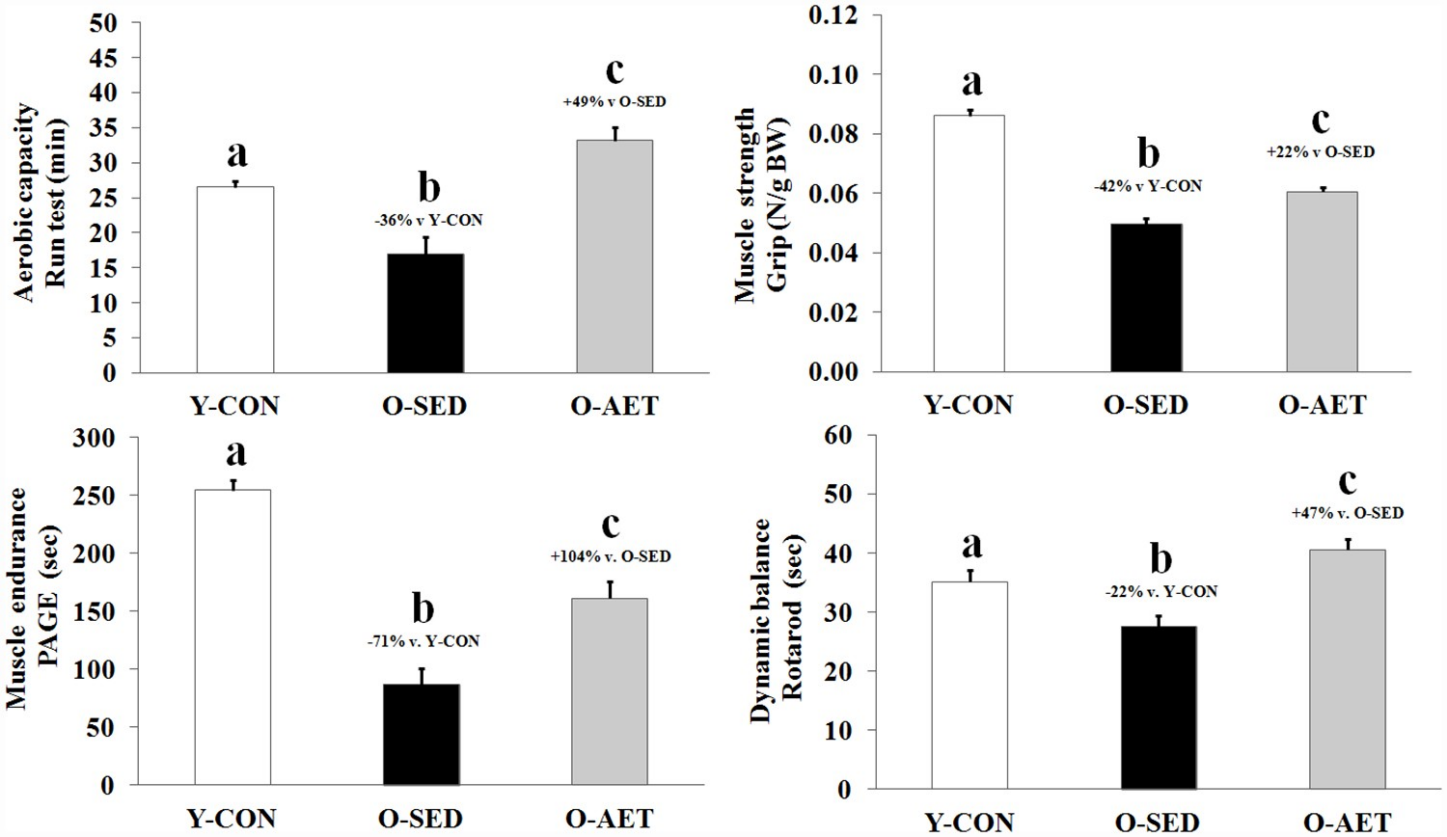
Lifelong aerobic exercise mitigates dynapenia and preserves aerobic fitness and motor function in old C57BL/J6 mice. **Top left)** Maximal aerobic capacity (min). **Top right)** Maximal forelimb grip-strength (N). **Bottom left)** Muscle endurance/grid-hang (PaGE; sec). **Bottom right)** Dynamic balance/motor coordination (Rotarod; sec). Complete data-sets are shown in S1 and S2 Tables. Group means (bars) that do not share the same alphabetical letter(s) are statistically different at P < 0.05.

Skeletal muscle (SM) comprises 30–50% of total bodyweight in mature adults and is lost at 3.7–4.7% of peak mass per decade between 18 and 80 years of age [63, 64]. Age-related muscle loss is classified as sarcopenia (‘flesh-deficiency’) when falling two standard deviations below the young adult mean [65], and is estimated to affect more than 50 million individuals worldwide [66]. Sarcopenia and obesity are independent risk factors of NCDs, and act synergistically to worsen prognostic outcomes and disability [67]. Analogous to the human condition, aging in C57BL/J6 mice is characterized by a relative increase in fat mass versus lean mass [68]. Consistent with these observations, the O-SED cohort exhibited significant bodyweight gain concurrent with SM wasting (Fig 1A and 1B), which primarily affected the fast-twitch muscles of the lower leg, including the quadriceps and anterior crural muscle groups (S2 Table). As shown previously [56], whole brain (specific regions not assessed), heart, liver, kidney, and spleen were enlarged with aging, while testicles underwent significant atrophy (Fig 1C). Skeletal muscle deterioration was coupled with reduced locomotion, aerobic deconditioning, lower muscle strength and endurance (dynapenia; ‘power deficiency’), and impaired motor coordination (Fig 2 and S1 Table). The disproportionate loss of functional capacity (-32%, -42%, and -71% in muscle mass vs. strength vs. endurance, respectively) likely reflects age-related degeneration of neural components (α-motor neurons, motor units etc.) which are integral for force/power production, and have previously been shown to contribute significantly to weakness and frailty at old age [63].

### Lifelong AET preserves organ reserves

A sedentary lifestyle is very common among elderly and a globally occurring phenomenon, regardless of geographic area and social development index (6). This age-related decrease in PA levels also appears to be conserved across many species on Earth (from worms to humans), and accelerates the rate of organ deterioration in a positive feedback manner. Predictably, regular PA maintains organ reserves and prolongs health- and lifespan, particularly in modern humans [9–15]. Widely recognized as the foundation of cardiometabolic disease prevention, aerobic exercise training (AET) protects against obesity, T2DM, and death from cardiovascular events by enhancing oxidative capacity and maximal aerobic power [69]. Additional benefits include pro-anabolic effects in SM, enhanced musculoskeletal integrity, and improvements in organs that are not classically associated with exercise (such as skin, kidney, and brain) [70]. Our results are in general agreement with those of Garcia-Valles and colleagues (2015), who demonstrated that lifelong wheel running prevented several indices of frailty and improved overall health in C57BL/J6 mice, while it did not enhance median or maximal lifespan significantly [58]. Specifically, we report that lifelong AET mitigated bodyweight gain, muscle loss, strength deficits, and testicular atrophy, and even elevated motor function and aerobic fitness above young control levels (Figs 1 and 2). However, despite many favorable adaptations predicted to extend lifespan, the survival rate at 26 months (i.e. median lifespan; equivalent to 70–75 human years) was only modestly improved in lifelong runners vs. sedentary controls (+6% vs. O-AET vs. O-SED; NS). Notwithstanding, a 3–10% extension in average life-expectancy is consistent with large population-based studies in human runners [19, 20].

### Aging and systemic inflammation

In parallel with age-related declines in organ reserves, glucose tolerance, and sex-hormones, systemic inflammatory factors steadily rise from the third decade onwards in humans [71, 72]. Imperfect repair, recycling, and removal of danger-associated molecular patterns (DAMPs) are postulated to trigger activation of evolutionary conserved cell danger response programs (CDR and DDR, respectively), which fuel a vicious cycle of unresolved cell damage, build-up of toxic debris, senescence, and inflammation [52]. Inflammaging is thereby multi-factorial, but principally attributed to DAMP-activation of the innate immune system, immunosenescence, and the senescence-associated secretory phenotype (SASP) [73].

Pro-inflammatory gerokines, such as TNF, IL-1, and IL-6, are positively correlated with morbidity and mortality in elderly [71, 74], and predispose to a wide spectrum of NCDs, including cardiovascular diseases, neurodegenerative disorders, and cancers [33–37, 42, 75, 76]. However, the biological effects of any cytokine *in vivo* depends on the presence of other cytokines with additive, synergistic, or antagonistic modes of action [77]. For example, IL-1 and TNF-α work in synergy to initiate and amplify the cytokine cascade, and blockade of either master regulator improves survival following infection [78, 79]. The remodeling theory of aging, as outlined by Franceschi et al., stipulates a progressive accumulation of both pro- and anti-inflammatory gerokines occurring simultaneously with aging [39, 41].

**Fig 3:**
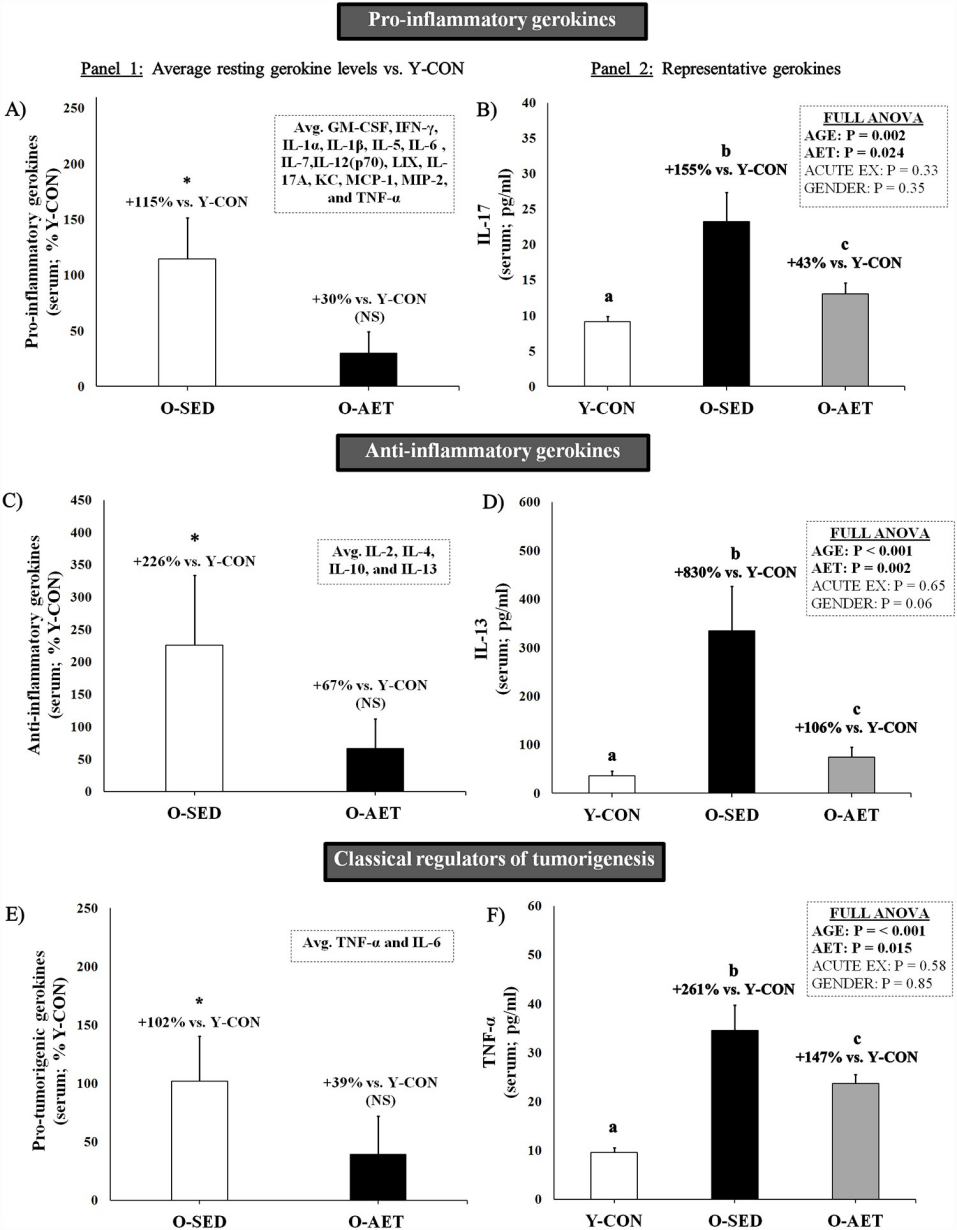
Aging and lifelong aerobic exercise effects on pro-inflammatory, anti-inflammatory, and pro-tumorigenic serum gerokines. **Panel 1)** Average resting serum gerokine levels in 26-mo-old sedentary (O-SED) and lifelong aerobically trained (O-AET) C57BL/J6 mice vs. 2-mo-old controls (% Y-CON). Acute exercise groups were omitted from these analyses. **Panel 2)** Representative serum gerokines (pg/mL) collapsed within main groups. Full data sets are available in the supplementary data section. For panel 1, NS = not significantly different from Y-CON. *Significantly higher vs. Y-CON (P ≤ 0.05). For panel 2, group columns that are significantly different do not share the same letter(s) (P ≤ 0.05).

In support of the remodeling theory, we found that a vast majority of pro- and anti-inflammatory serum markers were chronically elevated by aging (Fig 3A and 3C; pro-inflammatory: + 115% and anti-inflammatory: +226% vs. Y-CON), with a main effect of age in 17 of 18 targets (S3 and S4 Tables). The full ANOVA design indicated that IL-17a (Fig 3B; +155% vs. Y-CON), IL-13 (Fig 3D; +830%), TNF-α (Fig 3F; +261%), IFN-γ (+534%), IL1-β (+360%), GM-CSF (+262%), CCL-2/MCP-1 (+235%), and CXCL1/KC (+46.3%) were the most robust markers of inflammaging in the C57BL/J6 model. Notably, IL-1β and TNF-α, and to a lesser extent IL-6, were significantly elevated in O-SED as compared to Y-CON (Fig 3E and 3F), and are considered key drivers of the cytokine cascade and cancer progression [42, 43]. Other gerokines, such as IL-1α (+61%, IL-2 (+107%), IL-4 (+901%), IL-6 (+373%), IL-10 (+1006%), and IL-12(p70) (+1074%), also contributed to the overall inflammatory state in old mice, but statistical significance was partly driven by the acute exercise response in O-SED and O-AET groups.

**Fig 4:**
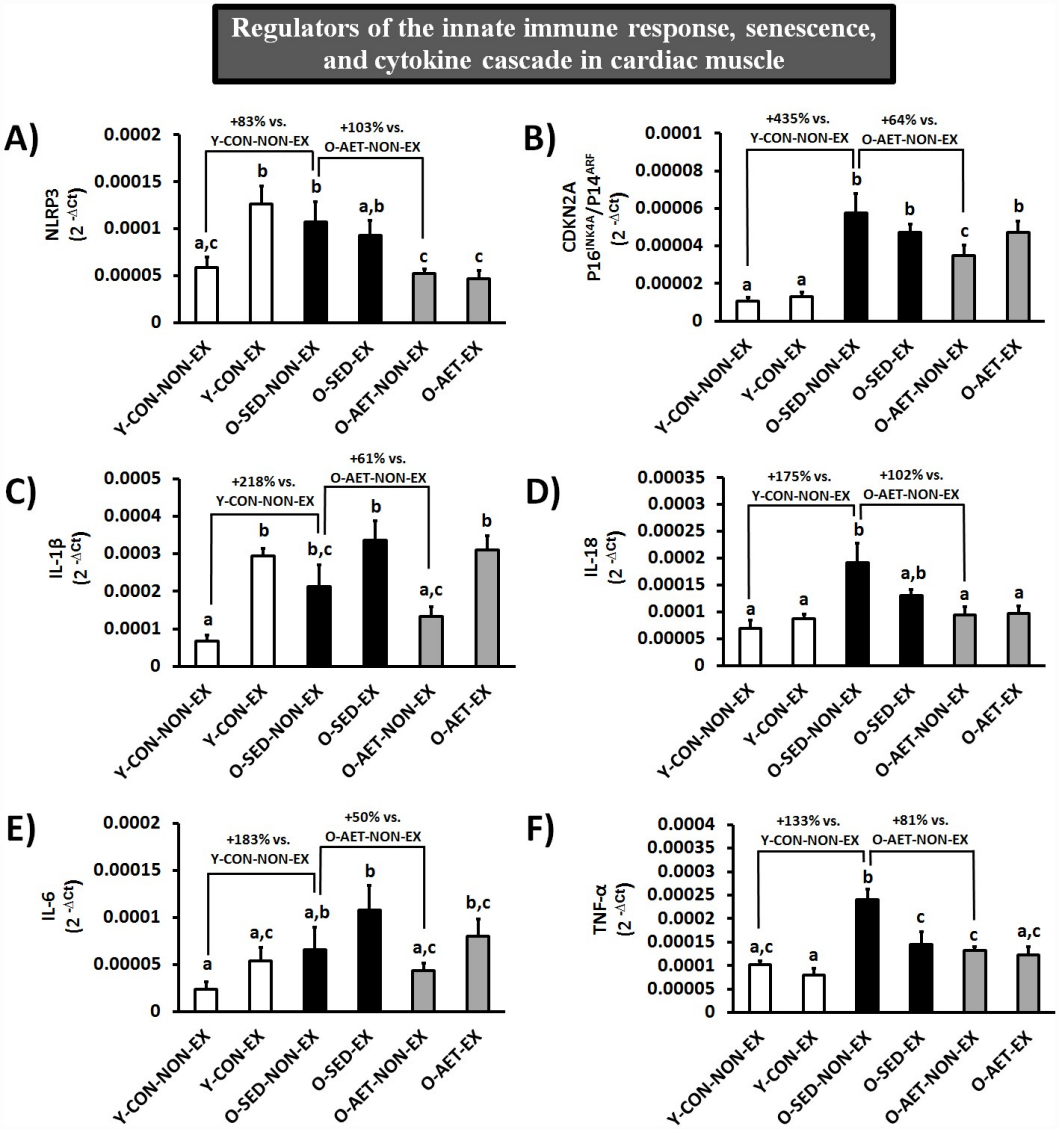
Cardiac muscle mRNA expression of regulators of the innate immune response and cell cycle progression. Group columns that are significantly different do not share the same letter(s) (P ≤ 0.05).

Although multiple tissue- and cell-types contribute to the aging secretome *in vivo*, we analyzed the expression of regulators of the innate immune system (NLRP3), cell-cycle progression (CDKN2A/P16^INK4A^), and the cytokine cascade (IL-1β, IL-6, TNF-α, and IL-18) in cardiac and skeletal muscles of young and old mice (Fig 4, S2 Fig, and S8 Table, respectively). Our findings largely support the contention that the innate immune system is constitutively primed and/or activated at old age, while the cell cycle is progressively inhibited (i.e. cellular senescence), both of which may contribute to inflammaging and SASP [73]. Specifically, basal mRNA levels of NLRP3 (+83%), CDKN2A (+435%), IL-1β (+218%), IL-18 (+175%), IL-6 (+183%), and TNF-α (+133%) were substantially elevated in unexercised O-SED vs. Y-CON mice (Fig 4A–4F).

### Aging and myokines

In principle, all cells that undergo exchange with the extracellular environment may contribute to the myriad of blood-borne factors that exert autocrine, paracrine, and endocrine effects. For example, immune, muscle, and fat cells each provide a unique spectrum of proteins to the circulatory system (cytokines, myokines, and adipokines, respectively), while also exhibiting significant overlap between their secretomes (e.g., myocytokines, adipocytokines, adipomyokines etc.).

Skeletal muscle may be considered the largest endocrine organ in the body that actively secretes myokines at rest and in response to various stressors (pathological, contractile, metabolic etc.). To date, over 300 factors secreted by primary SM cells have been identified using targeted and exploratory proteomic approaches [64, 80–82] including a substantial number of cytokines and chemokines (e.g., myocytokines; GM-CSF, IFN-γ, IL-1α/β, IL-2, IL-4, IL-5, IL-6, IL-7, IL-8, IL-10, IL-12 (p70), IL-13, IL-15, IL-17A, CXCL1/KC, LIF, CCL2/MCP-1, and TNF-α). Many other secretory proteins, such as osteonectin (SPARC), ostecrin (musclin), follistatin-like protein 1 (FSTL-1), myostatin (MSTN), FGF-21, fractalkine (CXC3CL1), VEGF-A, BDNF, metorin-like (Metrnl), and irisin, are either confirmed myokines or promising candidates.

Aging has well-documented adverse effects on SM cells, including build-up of DAMPs, chronic immune activation, and myofibrillar atrophy, all of which may alter the muscle-specific secretome. Resident and invading immune cells, adipocytes, and fibroblasts, also contribute to the SM secretory spectrum *in vivo*, especially during remodelling and pathological processes. Because muscle fiber degeneration may fuel systemic inflammation independently (in muscular dystrophies, for example), it is likely that age-related SM deterioration partly underlies inflammaging. Regardless of the tissue/cell origins, the key drivers of the cytokine cascade, TNF-α, IL-1β, and IL-6 (+128%, +159%, and +75%, respectively, O-SED vs. Y-CON), were chronically elevated at old age, and may have contributed to SM wasting in this study. Further indicative of anabolic dysregulation and an overall pro-catabolic systemic environment, myostatin, follistatin-like protein 1, FGF-21, and osteocrin/musclin, and to a lesser extent IL-15 and fractaline/CX3CL1, were significantly lower in O-SED vs. Y-CON cohorts (S5 and S6 Tables).

**Fig 5:**
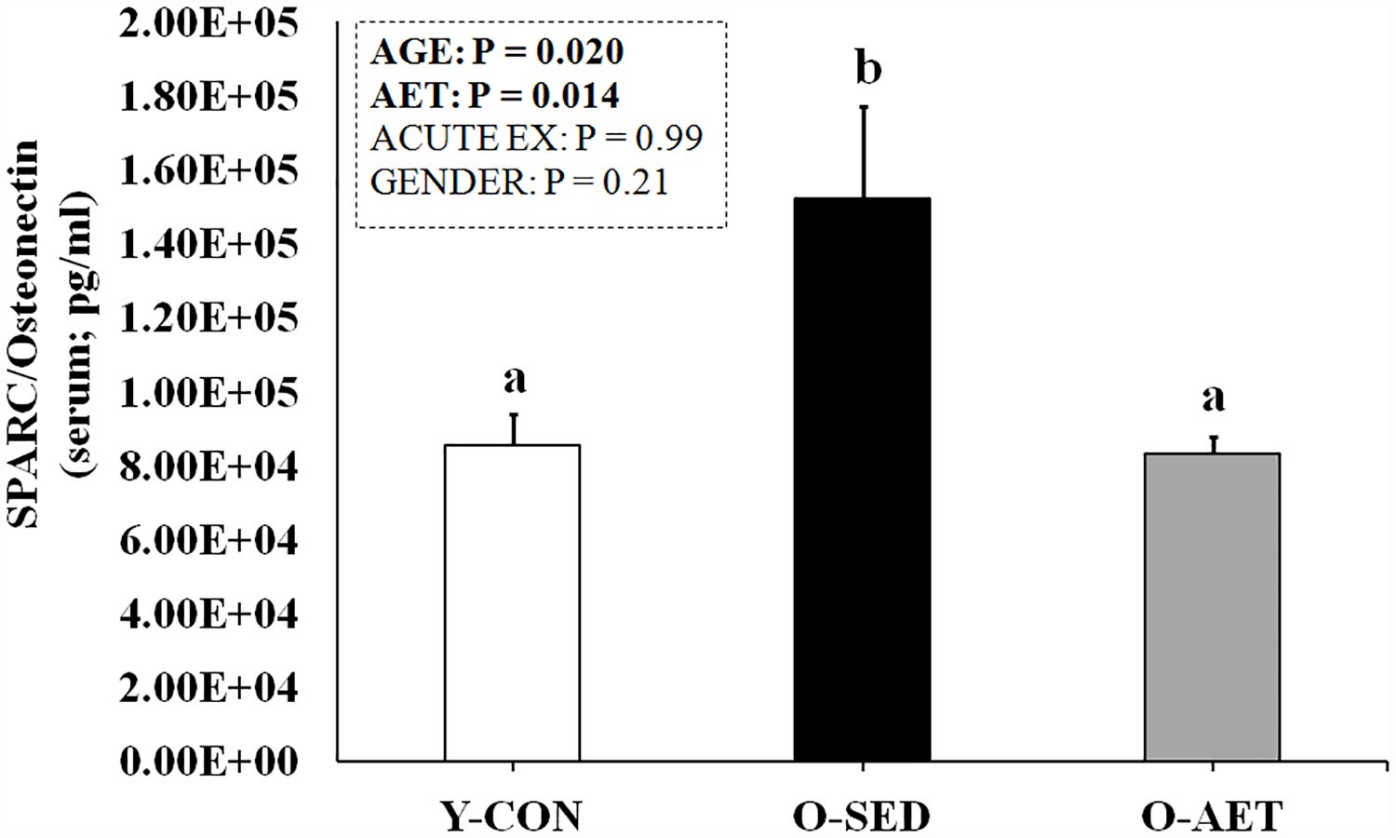
Basal serum levels of SPARC in 26-mo-old sedentary (O-SED) and lifelong aerobically trained (O-AET) C57BL/J6 mice vs. 2-mo-old controls (% Y-CON). Group columns that are significantly different do not share the same letter(s) (P ≤ 0.05).

Conversely, the matricellular protein SPARC/osteonectin followed the same general pattern as pro- and anti-inflammatory gerokines and was elevated with aging (Fig 5). SPARC is expressed during remodelling and repair in intestinal, glandular, skin, muscle (cardiac, smooth and skeletal), adipose, and neoplastic tissues [83]. In aggressive tumor phenotypes, SPARC may act as a positive regulator of metastatic potential by augmenting vascular permeability [84]. Persistent induction of SPARC promotes tissue fibrosis, nephropathy, retinopathy, and non-alcoholic fatty liver disease, while its deletion/inhibition protects against the same [85–87]. Considering that circulating SPARC is up-regulated in metabolic disease, accelerated aging conditions (Werner Syndrome etc.), and in sarcopenia [85, 88–93], it is conceivable that it may be used as a general biomarker of advancing age and/or pathological aging processes.

### Lifelong AET mitigates inflammaging

In contrast to chronic stress, the intermittent application of a physiological challenge, such as AET, minimizes perturbations to the intracellular milieu and allows for sufficient recovery for the cell to evolve into a more stress-resistant, homeostatic level, thereby attenuating biological aging and protecting against inflammation [51, 52]. The anti-inflammatory effects of exercise training are well-documented (with higher IL-10, and concomitantly lower TNF-α, IL-6 and CRP levels), and benefits appear to be independent of age and chronic disease [94]. As expected, lifelong AET effectively dampened inflammaging in the C57BL/J6 model, with a restoration of both pro- and anti-inflammatory gerokines to young control levels (Fig 3A and 3C; NS vs. Y-CON), including the pivotal regulators of the cytokine cascade and tumor development (Fig 3E; NS vs. Y-CON). In the full ANOVA design, 10 out of 17 age-induced markers were AET-responsive, specifically IL-17a (Fig 3B), IL-13 (Fig 3D), TNF-α (Fig 3F), IL-6, GM-CSF, IFN-γ, IL-2, IL-10, IL-12 (p70), and CCL-2/MCP-1 (S3 and S4 Tables). Although a majority of gerokines were significantly attenuated and even normalized by lifelong running (notably IL-13, GM-CSF, IFN-γ, IL-17, TNF-α, and IL-1β), basal levels of IL-10 and CXCL1/KC were moderately enhanced by AET (+25% vs. O-SED).

IL-10 inhibits the activity of TH1 cells, NK cells, and macrophages and the production of wide range of pro-inflammatory cytokines and chemokines, including TNFα, IL-1α/β, IL-6, and MCP-1 [95]. By counteracting the proteolytic effects of IL-1β, the anti-inflammatory actions of IL-10 are integral for muscle repair and growth following strenuous exercise [96]. CXCL1/KC (GRO-α) is a chemoattractant that stimulates neutrophil recruitment and phagocytosis of injured tissue, but the functional significance of elevated serum KC levels following endurance training is unclear. Overexpression of CXCL1/KC in SM promotes its systemic release and induces an oxidative phenotype in high-fat fed mice, which protects against fat gain and glucose intolerance [97]. CXCL1/KC may therefore play an integral role in mediating the whole-body metabolic benefits of aerobic exercise training and be of therapeutic interest as an exercise mimetic. Lastly, circulating SPARC/osteonectin was restored to young control levels by lifelong running (Fig 5), further suggestive of SPARC being a general biomarker of advancing age and/or pathological aging. We did not observe any significant effects of lifelong AET on basal FSTL-1, musclin, or fractalkine; while MSTN (↑), FGF-21 (↓) and IL-15 (↓) were moderately affected in our study (S5 and S6 Tables).

### Lifelong AET alters the inflammatory response to acute exercise

Aerobic exercise is a single, hormetic stress stimulus that encompasses transitory spikes in intracellular danger signals (Ca^2+^, ROS, pH, and hypoxia), metabolic intermediates (NAD+/NADH and AMP/ATP), and a myriad of neurotransmitters, hormones, and organokines, which act synchronously to stimulate mitochondrial biogenesis, antioxidant defense, cellular repair and recycling, and immunity [45–50]. We recently coined the term ‘exerkine’ to describe any biomolecule released into circulation in response to exercise, including non-coding RNAs, mRNAs, metabolites, peptides, and proteins [49]. Approximately 50% of all SM-derived secretory factors are contractile activity-mediated [64], and other cell types likely contribute significantly to the total exerkine pool *in vivo*, principally liver, fat, and immune cells [77].

**Fig 6:**
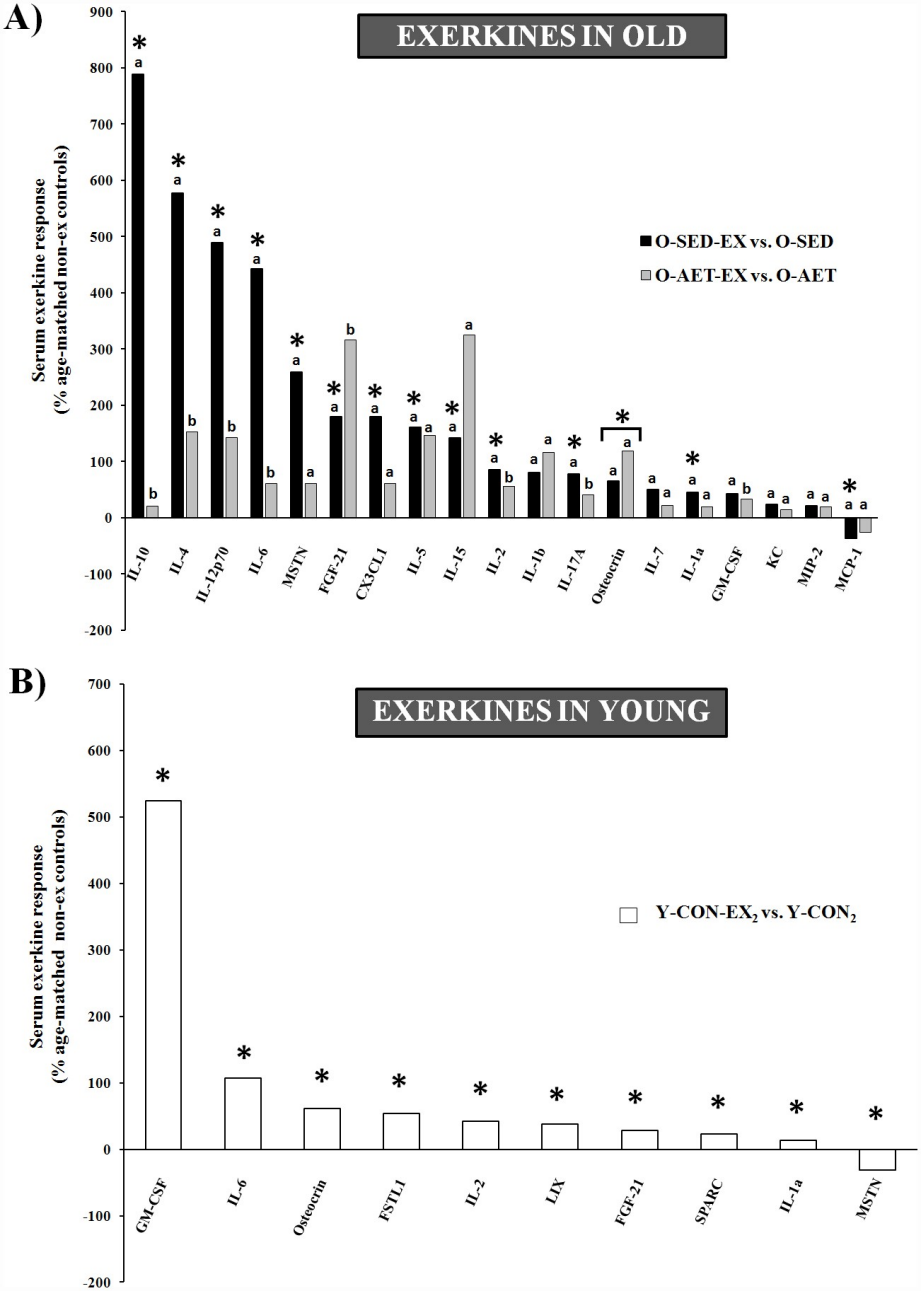
Contractile activity-induced chemokines, cytokines, and myokines (‘exerkines’) in serum of young and old C57BL/J6 mice. **A)** Acute exercise response of sedentary (O-SED-EX; black bars) and life-long trained (O-AET-EX; gray bars) C57BL/J6 mice expressed relative to unexercised, age-matched controls (O-SED vs. O-AET, respectively). Main effects of exercise were mainly attributed to old cohorts as shown above and in Table 1a (left column). By removing young animals from the statistical analyses (Table 1b middle column), several additional exerkines were uncovered in old mice. **B)** Maximal exercise response of young C57BL/J6 mice expressed relative to unexercised controls (Y-CON-EX_2_ vs. Y-CON_2_; Table 1c right column). *Significantly different from unexercised, age-matched controls (P ≤ 0.05). Groups that do not share the same letter(s) are significantly different (P ≤ 0.05).

Several basic parameters may affect the systemic response to acute contractile activity, such as exercise variables (e.g., mode, intensity, and duration), blood sampling (time and fraction), training status, gender, and age. As reviewed by Peake at el., the exercise-mediated increase in circulating cytokines is highly variable and typically reflects the relative exercise intensity and degree of intracellular stress/damage [77]. In our study, an acute bout of aerobic exercise elicited a significantly higher inflammatory response in old sedentary C57BL/J6 mice (O-SED-EX) compared to aerobically trained (O-AET-EX) and young counterparts (Y-CON-EX), suggestive of a protective adaptation induced by lifelong AET to intracellular stress (S3 and S4 Tables). Statistical significance was almost exclusively driven by the robust exercise effect in O-SED-EX (AGE x ACUTE EXERCISE x GENDER; Table 1a) and included many classical pro- and anti-inflammatory cytokines previously identified as exercise factors. To illustrate this further, we analyzed the secretory response from old cohorts independently (AET x ACUTE EXERCISE x GENDER; Table 1b), and observed that the vast majority of inflammatory factors (14/18) and myokines (5/7) were contractile activity-regulated at old age (Fig 6A). A second cohort of young animals was vigorously exercised until complete exhaustion (Y-CON-EX_2_), and this intense physiological stimulus was insufficient to induce the same magnitude and breadth of cytokines as in O-SED (Fig 6B; Table 1c; S7 Table). Other notable age discrepancies in the secretory response were SPARC (↑ young vs. ↔ old), FSTL-1 (↑ young vs. ↔ old), and MSTN (↓ young vs. ↑ old), while musclin and FGF-21 were elevated by acute exercise regardless of age (Table 1b and 1c).

**Table 1:** Contractile activity-induced cytokines, chemokines, and myokines (‘exerkines’) in serum of young and old C57BL/J6 mice.

A) ANOVA 1: [age * acute ex * gender]			B) ANOVA 2: [AET* acute ex * gender]			C) Independent T-tests		
	YOUNG AND OLD			OLD			YOUNG	
NAME	P-VALUES	DIRECTION	NAME	P-VALUES	DIRECTION	NAME	P-VALUES	DIRECTION
***Cytokines and chemokines***			***Cytokines and chemokines***			***Cytokines and chemokines***		
IL-10	P_EX_ = 0.033	↑	IL-10	P_EX_ = 0.016	↑	IL-6	P < 0.001	↑
IL-4	P_EX_ = 0.050	↑	IL-4	P_EX_ = 0.015	↑	IL-2	P = 0.055	↑
IL-12	P_EX_ = 0.014	↑	IL-12	P_EX_ = 0.002	↑	IL1α	P = 0.005	↑
IL-6	P_EX_ = 0.024	↑	IL-6	P_EX_ = 0.007	↑	GM-CSF	P = 0.005	↑
IL-2	P_EX_ = 0.068	↑	IL-2	P_EX_ < 0.001	↑	LIX	P < 0.001	↑
IL1α	P_EX_ = 0.013	↑	IL1α	P_EX_ = 0.003	↑	***Myokines***		
KC	P_EX_ = 0.009	↑	KC	P_EX_ = 0.030	↑	OSTN	P = 0.038	↑
MIP-2	P_EX_ = 0.019	↑	MIP-2	P_EX_ = 0.008	↑	FGF-21	P = 0.006	↑
MCP-1	P_EX_ = 0.001	↓	MCP-1	P_EX_ = 0.014	↓	Myostatin	P = 0.002	↓
***Myokines***			GM-CSF	P_EX_ = 0.023	↑	FSTL1	P = 0.00	↑
OSTN:	P_EX_ = 0.070	↑	IL-1β	P_EX_ = 0.012	↑	SPARC	P < 0.001	↑
			IL-5	P_EX_ = 0.011	↑			
			IL-7	P_EX_ = 0.004	↑			
			IL-17	P_EX_ = 0.033	↑			
			***Myokines***					
			OSTN	P_EX_ = < 0.001	↑			
			IL-15	P_EX_ = < 0.001	↑			
			FGF-21	P_EX_ = 0.002	↑			
			Myostatin	P_EX_ <0.001	↑			
			Fractalkine	P_EX_ <0.001	↑			

**A)** Exerkines according to 2 x 2 x 2 ANOVA across age groups (AGE x ACUTE EXERCISE x GENDER). **B)** Exerkines according to 2 x 2 x 2 ANOVA in old mice (AET x ACUTE EXERCISE x GENDER) (Fig 6A). **C)** Exerkines according to an additional cohort of young C57BL/J6 mice exercised until exhaustion and analyzed by independent t-tests (Fig 6B). The arrows depict the directionality of the contractile activity-induced response of each exerkine.

### Lifelong AET protects against cancer

Inflammaging predisposes for a wide range of NCDs, including multi-systemic cancers. Chronic inflammation directly underlies ~20% of all cancers and a vast majority of solid malignancies contain inflammatory cells that secrete cytokines and chemokines, which can modulate all stages of tumor development (e.g., initiation, promotion, malignant conversion, invasion, and metastasis) [42, 43]. Macrophages (TAMs) and T-cells (CD8^+^cytotoxic, CD4^+^ helper, and NKT) are the most abundant immune cells in the tumor microenvironment, and pro-inflammatory cytokines enable dynamic cross-talk between cell populations [42]. In over 50% of all cancers, apoptotic resistance and high proliferative rates are attributed to aberrant NF-κB, AP-1, and STAT3 signalling caused by the autocrine and paracrine actions of TNF-α and IL-6 [43].

The anti-tumorigenic effects of regular PA may be attributed to attenuating the loss of organ reserves, DAMP removal, and mitigation of the pro-inflammatory, systemic environment. Acutely, aerobic exercise is a stress stimulus that induces a CDR-driven inflammatory response, which is rapidly resolved and favors tumor clearance [98]. Specifically, voluntary wheel running has previously been shown to inhibit cancer development across a range of tumor models in mice, and involves an epinephrine-dependent mobilization of IL-6Rα-positive NK cells that require IL-6 for efficient tumor homing and infiltration [32].

**Fig 7:**
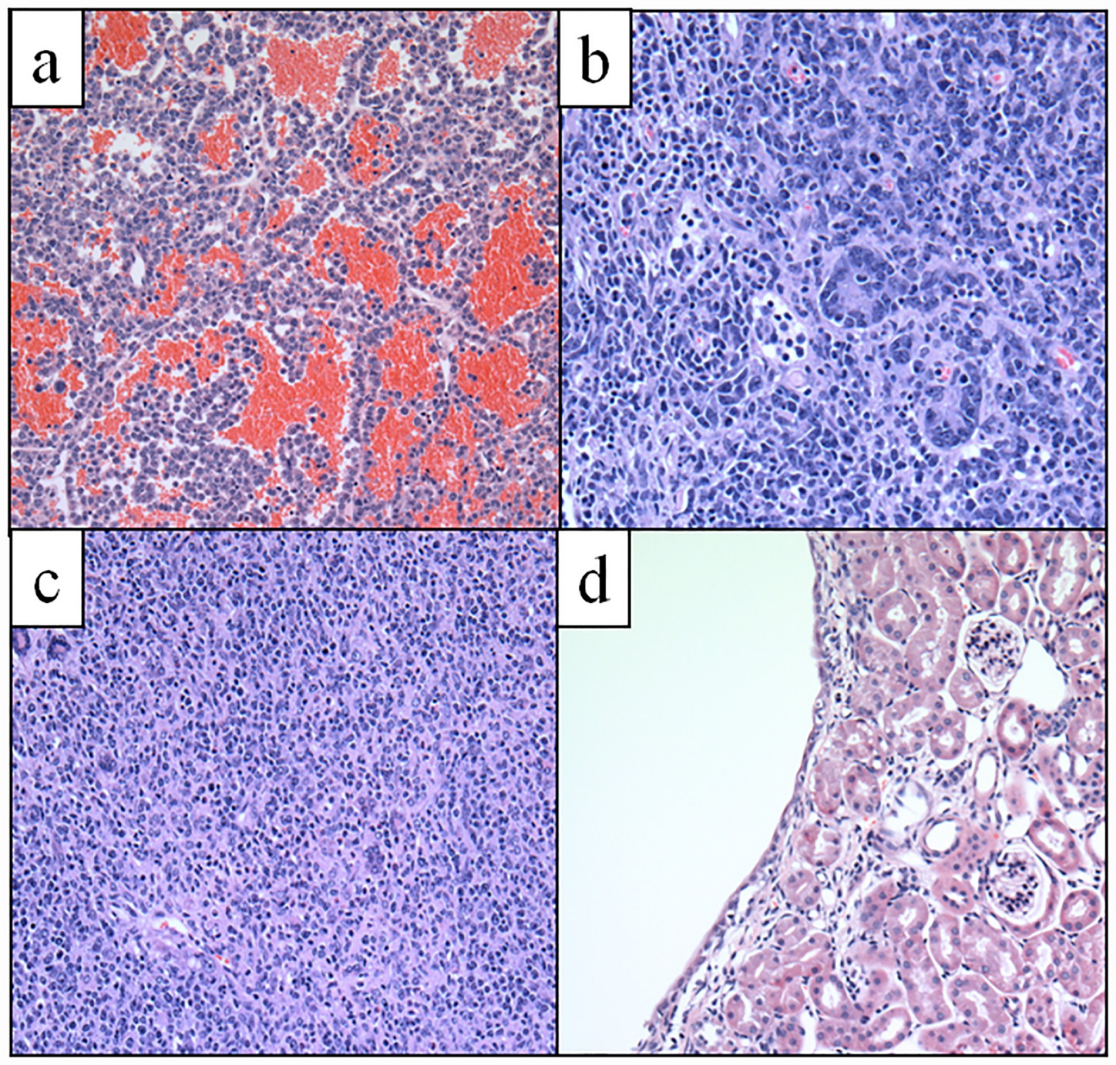
Examples of tumor histopathology. **A)** Pituitary neoplasm, **B)** Intestinal adenocarcinoma, **C)** Hematopoietic neoplasia, and **D)** Kidney: Benign renal cysts. H&E stain; 200X.

**Fig 8:**
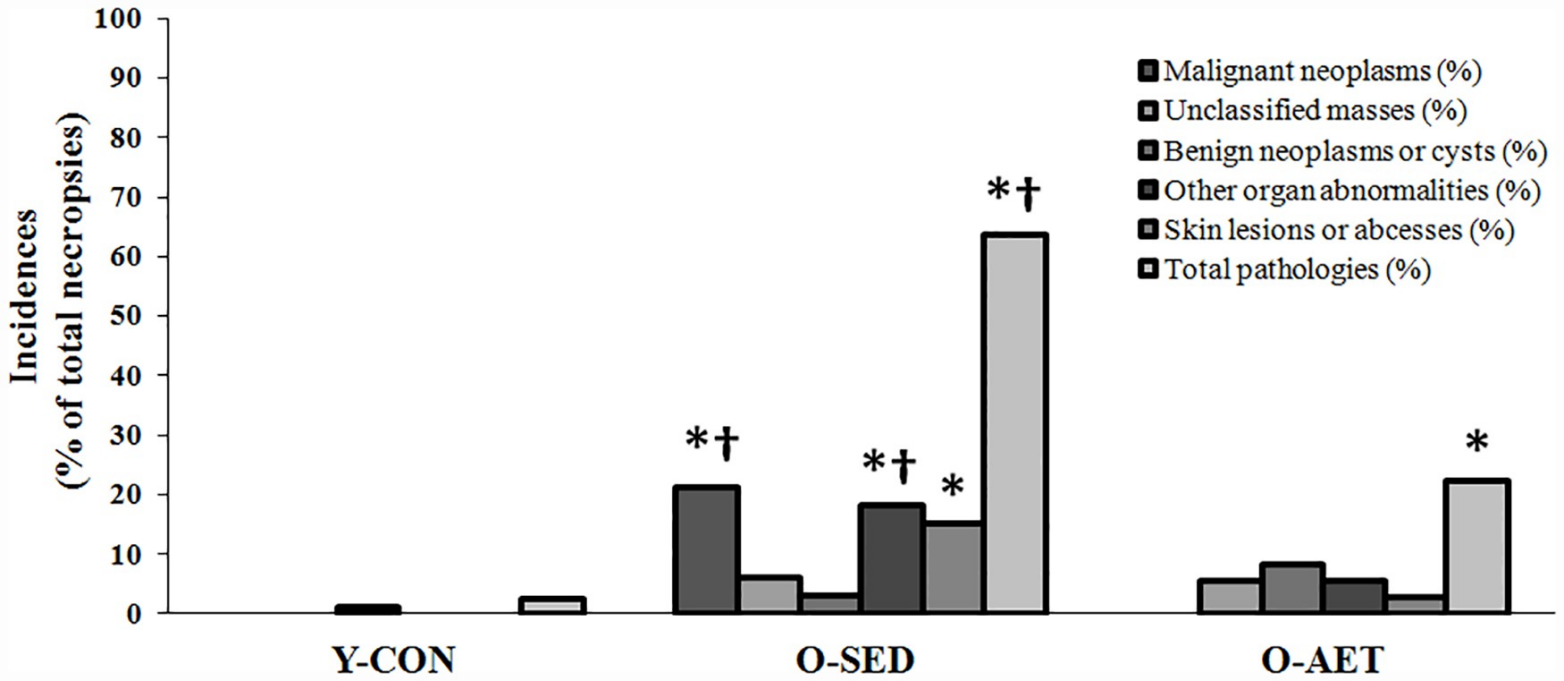
Incidences of multi-system cancers, benign cysts, unclassified masses, skin pathologies, and other organ abnormalities in young control (Y-CON), old sedentary (O-SED), and lifelong aerobically trained (O-AET) C57BL/J6 mice. Pathology frequencies in Table 1 were coded as binary data (present vs. non-present), evaluated by either Chi-square or Fisher’s Exact tests, and graphed as % incidences for each group [(total frequency of pathology/number of necropsies)*100)]. Symbols indicate significantly higher (P < 0.05) vs. Y-CON (*) or Y-CON and O-AET (*†).

In this study, we found malignant tumors in the liver (N = 3), brain (pituitary; N = 2), intestines (N = 1), and spleen (N = 1) of O-SED C57BL/J6 mice, while lifelong runners were cancer-free (Table 2). In keeping with the Jackson Laboratory Mouse Tumor Biology Database [99], we report that organs most likely to become malignant were the liver, pituitary, and intestines, and that these tumors were mainly hematopoietic malignancies (e.g., lymphomas) and adenocarcinomas (Fig 7A–7C). In contrast to the exclusive finding of malignant tumors in the O-SED cohort, there was a roughly equal distribution of benign tumors in the reproductive (ovary and testicle), digestive (gallbladder and intestine), and urinary (kidney, Fig 7D) systems of non-runners vs. runners. Overall, lifelong running protected against total organ pathology (22% vs. 64% in O-AET vs. O-SED, respectively), multi-systemic cancers (0% vs. 21%), and skin deterioration (3% vs. 15%) (Fig 8).

**Table 2:** Frequency of cancers, benign cysts, unclassified mass, skin disease, and other organ pathology in young control (Y-CON), old sedentary (O-SED), and lifelong aerobically trained (O-AET) C57BL/J6 mice.

Cancers, cysts, or other organ pathology*	**Necropsies**	**Brain**	**Liver**	**Gallbladder**	**Kidney**	**Spleen**	**Intestine**	**Ovary**	**Testicle**
**Y-CON**	40	0	0	0	1	0	0	0	0
**O-SED**	33	2	4	0	1	3	1	3	2
**O-AET**	36	0	1	2	0	1	1	2	0
Skin pathology	**Lesions**	**Abscesses**	**Total**						
**Y-CON**	0	0	0						
**O-SED**	4	1	5						
**O-AET**	1	0	1						
Total pathologies	**Cancers**	**Unclassified mass**	**Benign cysts**	**Skin**	Other*	**Total**			
**Y-CON**	0	0	1	0	0	1			
**O-SED**	7	2	1	5	6	21			
**O-AET**	0	2	3	1	2	8			

*Other organ pathology included non-physiological organomegaly and atrophy not associated with neoplasms or cysts.

### Lifelong AET extends healthspan

**Fig 9:**
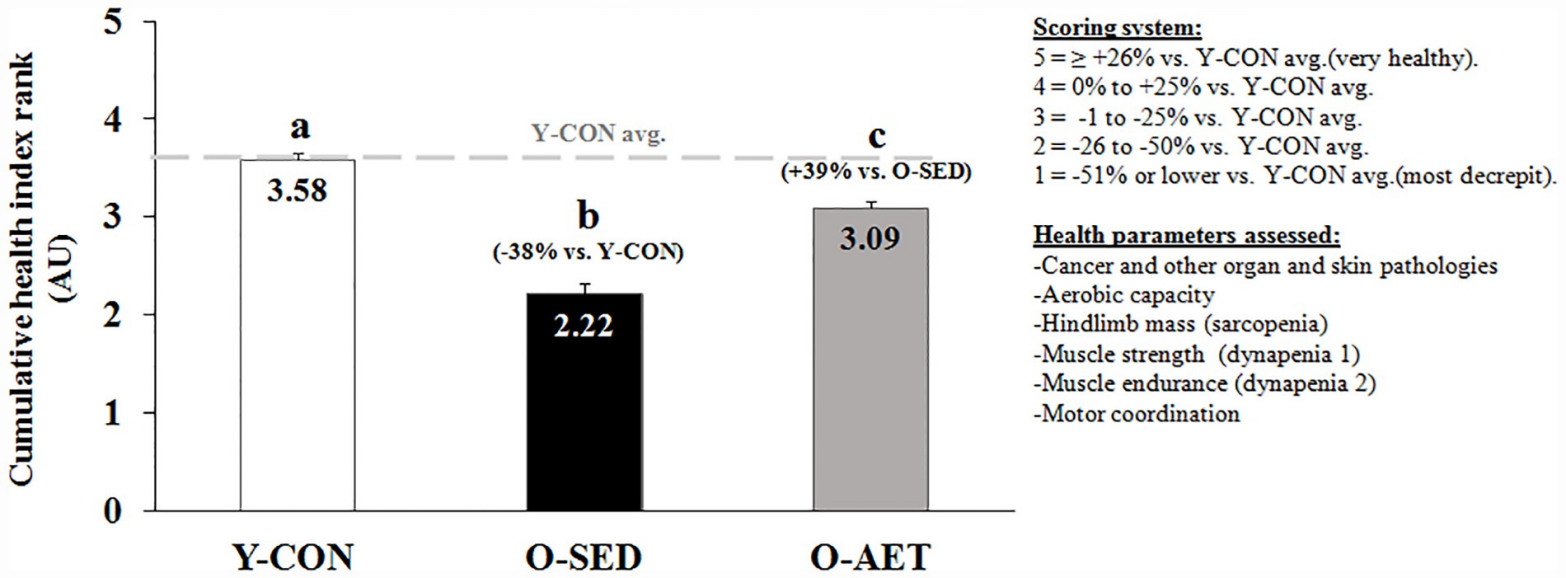
Cumulative health index rank in young control (Y-CON), old sedentary (O-SED), and lifelong aerobically trained (O-AET) C57BL/J6 mice. Common predictors of independence and health- and lifespan, including existing morbidities, aerobic capacity, skeletal muscle mass, muscle strength and endurance, and motor coordination were individually ranked on a 5-grade scale (see Methods), averaged and presented as a cumulative health index, and evaluated by Kruskal Wallis and Dunn’s multiple comparison tests. Group means that are statistically different from each other do not share the same alphabetical letter (P < 0.05).

**Fig 10:**
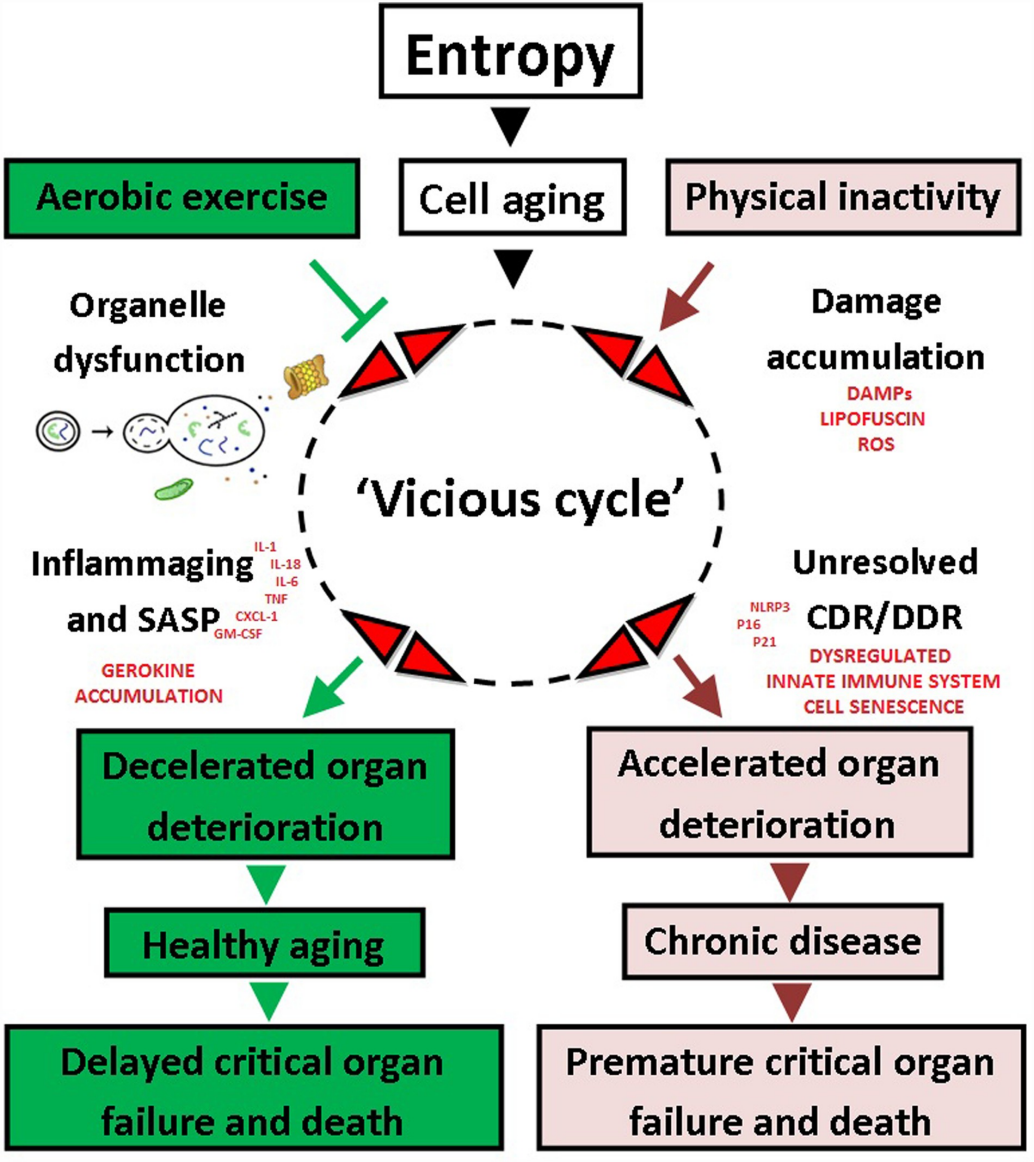
Integrated hypothesis of aging. Aging is inevitable and is characterized by progressive accumulation of damage from the 3^rd^-4^th^ decade and onwards in humans. Physical inactivity accelerates the cellular aging process in a ‘vicious cycle’ and predisposes for a wide spectrum of chronic diseases, including cancers. Aerobic exercise training (AET) mitigates inflammaging, reduces all-cause mortality risk, and extends health- and lifespan.

Cell aging is characterized by progressive malfunctioning of pivotal systems that regulate energy production, growth, and quality control (repair, folding, recycling/degradation, and synthesis), progressively depleting organ reserves and eventually causing death. Physical inactivity may accelerate this process by antagonizing a ‘vicious cycle’ of unresolved damage, persistent CDR/DDR activation, and inflammation. In this study, we found that early onset, lifelong AET dampened inflammaging, including master cytokines IL-1, TNF-α, and IL-6 (in circulation and tissue-specific), protected against multi-systemic cancers, and significantly enhanced healthspan of C57BL/J6 mice (cumulative health-index rank; Fig 9). These data support the contention that lifelong running decelerates the ‘vicious cycle’ and protects against the most salient features of the aging process, namely critical organ deterioration/pathology, frailty, and premature death (Fig 10).

## Conclusions

Cumulative evidence over the last 50 years unequivocally shows that sedentary living accelerates organ deterioration and increases all-cause mortality risk. In contrast, aerobic exercise training promotes healthy aging and extends average life expectancy by 3–10% [20]. Our data confirm that lifelong running provides significant health benefits and protects against cancer, while only modestly improving the median lifespan of naturally-aged mice. For more substantial pro-longevity benefits ‘of mice and men’, a combination of lifestyle modification, rejuvenation biotechnology, and intermittent pharmacotherapy may be necessary (e.g., anti-aging polytherapy). Given that the global burden of chronic disease is steadily rising (population aging and physical inactivity pandemic), the development of innovative strategies to increase daily PA levels at the societal level, and/or alternative means to harness the multi-systemic benefits of exercise (e.g., exercise mimetics), will be of paramount importance in the 21^st^ century.

## Supporting information

S1 Fig Voluntary running-wheel distances. Voluntary running-wheel distances decreased with aging in C57BL/J6 mice. Groups that do not share the same letter(s) are statistically different (P ≤ 0.05).
(DOC)

S2 Fig Quadriceps mRNA expression of regulators of the innate immune response and cell cycle progression. Group columns that are significantly different do not share the same letter(s) (P ≤ 0.05).
(TIFF)

S1 Table Functional data. Lifelong aerobic exercise training (AET) mitigates age-associated dynapenia and aerobic deconditioning in C57BL/J6 mice. *Significant effects of aging, †lifelong aerobic exercise training, and ‡gender (P ≤ 0.05).
(DOC)

S2 Table Morphological data. Lifelong aerobic exercise training (AET) mitigates age-associated muscle loss and testicular atrophy in C57BL/J6 mice. Age-associated organ wasting was isolated to fast-twitch muscles and testicles (test.), while slow-twitch muscles (solues; sol.) and other internal organs (heart, brain, liver, kidney, spleen, and lungs) were largely spared or increased in mass. Major thigh (quadriceps complex) and lower leg (anterior and posterior crural) muscles were summed to obtain total hindlimb muscle (HL) mass. *Significant effects of aging, †lifelong aerobic exercise training, and ‡gender (P ≤ 0.05).
(DOC)

S3 Table Cytokine and chemokine data. Lifelong aerobic exercise training (AET) dampens inflammaging in old C57BL/J6 mice. Serum cytokine and chemokine concentrations are reported in pg/mL and expressed as group means ± SE. Each sample consisted of serum from one or two mice within the same experimental condition. All samples were run in duplicate.
(DOC)

S4 Table 3-way ANOVA table for cytokine and chemokine data. (DOC)

S5 Table Myokine data. Lifelong aerobic exercise training (AET) mitigates serum SPARC levels in old C57BL/J6 mice. Serum myokine concentrations are in pg/mL and expressed as group means ± SE. Each sample consisted of serum from one or two mice within the same experimental condition. All samples were run in duplicate.
(DOC)

S6 Table 3-way ANOVA table for myokine data. (DOC)

S7 Table Serum exerkines in acutely exercised, young C57BL/J6 mice (Y-CON_2_ vs. Y-CON-EX_2_). Serum exerkine concentrations are reported in pg/mL and expressed as group means ± SE. Each sample consisted of serum from one or two mice within the same experimental condition. All samples were run in duplicate.
(DOC)

S8 Table Quantitative PCR methods. (DOCX)
