# Correction: Lifelong aerobic exercise protects against inflammaging and cancer

**DOI:** 10.1371/journal.pone.0233401

**Published:** 2020-05-13

**Authors:** Mats I. Nilsson, Jacqueline M. Bourgeois, Joshua P. Nederveen, Marlon R. Leite, Bart P. Hettinga, Adam L. Bujak, Linda May, Ethan Lin, Michael Crozier, Daniel R. Rusiecki, Chris Moffatt, Paul Azzopardi, Jacob Young, Yifan Yang, Jenny Nguyen, Ethan Adler, Lucy Lan, Mark A. Tarnopolsky

S9 Table is omitted from the list of Supporting Information. It can be viewed below.

## Supporting information

S9 Table LifeLong I Heart mRNA. Raw CT mRNA data.
(XLSX)
