# Supplementary figures and images for: Lifelong aerobic exercise protects against inflammaging and cancer
Source: PLoS One. 2019 Jan 25;14(1):e0210863. doi: 10.1371/journal.pone.0210863 (PMC6347267; doi:10.1371/journal.pone.0210863)

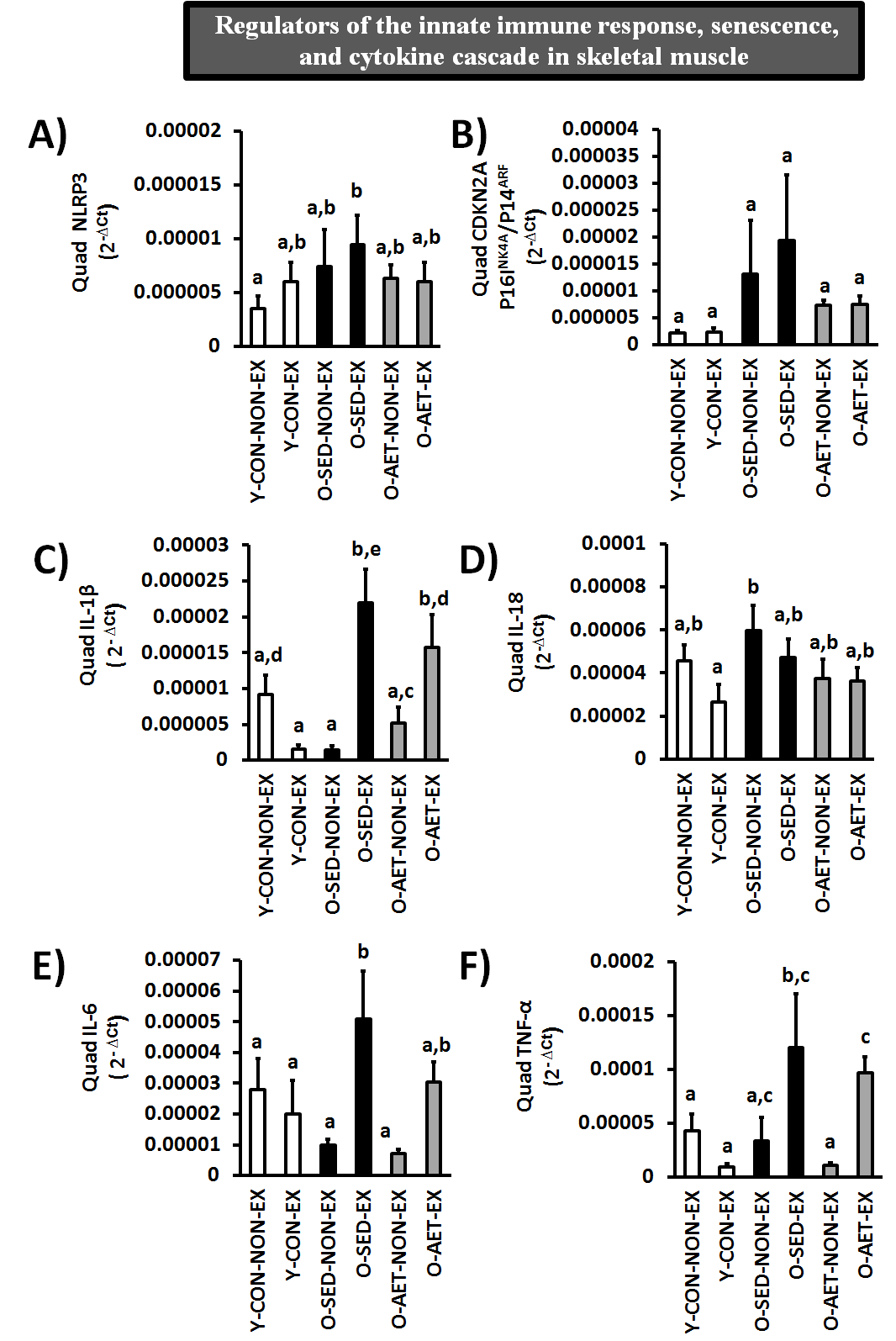

Supplement: S2 Fig — Group columns that are significantly different do not share the same letter(s) (P ≤ 0.05). (TIFF) [file pone.0210863.s002.tiff]
