# Supplementary material for: Lifelong aerobic exercise protects against inflammaging and cancer
Source: PLoS One. 2019 Jan 25;14(1):e0210863. doi: 10.1371/journal.pone.0210863 (PMC6347267; doi:10.1371/journal.pone.0210863)
Supplement: S1 Table — Lifelong aerobic exercise training (AET) mitigates age-associated dynapenia and aerobic deconditioning in C57BL/J6 mice. *Significant effects of aging, †lifelong aerobic exercise training, and ‡gender (P ≤ 0.05). (DOC) [file pone.0210863.s003.doc]

| **Group** | **Ambulation  (XAMB; X-axis)**  ***** | **Food intake (g)**  **†‡** | **RER**  **(VCO2/VO2)**  ***†‡** | **Aerobic capacity**  **(min)**  ***†** | **Grip strength (N/g BW)**  ***†‡** | **Muscular endurance**  **(PAGE; s)**  ***†‡** | **Dynamic balance (RotaRod; s)**  ***†** |
| --- | --- | --- | --- | --- | --- | --- | --- |
| ***Y-CON***  *(N = 40; 2-mo-old)* |  |  |  |  |  |  |  |
| M (20) | 387.54 ± 65.34 | 2.88 ± 0.28 | 0.94 ± 0.0085 | 24.62 ± 0.90 | 0.083 ± 0.0021 | 219.15 ± 14.12 | 37.56 ± 3.38 |
| F (20) | 437.61 ± 79.68 | 2.88 ± 0.30 | 0.95 ± 0.0078 | 28.66 ± 2.50 | 0.090 ± 0.0029 | 289.70 ± 4.83 | 32.80 ± 1.51 |
| ***O-SED***  *(N = 32; 26-mo-old)* |  |  |  |  |  |  |  |
| M (18) | 310.49 ± 47.11 | 2.59 ± 0.22 | 0.88 ± 0.0079 | 15.11 ± 1.90 | 0.043 ± 0.0019 | 39.39 ± 5.19 | 27.49 ± 2.25 |
| F (14) | 319.34 ± 36.74 | 3.10 ± 0.29 | 0.94 ± 0.0095 | 19.22 ± 2.65 | 0.058 ± 0.0030 | 147.79 ± 21.97 | 27.54 ± 3.22 |
| ***O-AET***  *(N = 38; 26-mo-old)* |  |  |  |  |  |  |  |
| M (18) | 266.83 ± 33.42 | 3.17 ± 0.35 | 0.94 ± 0.0076 | 29.96 ±2.56 | 0.057 ± 0.0021 | 91.94 ± 13.51 | 37.82 ± 2.64 |
| F (20) | 374.46 ± 48.35 | 3.46 ± 0.28 | 0.93 ± 0.0074 | 36.06 ± 4.13 | 0.063 ± 0.0023 | 224.05 ± 14.94 | 43.03 ± 2.43 |
