# Supplementary material for: Lifelong aerobic exercise protects against inflammaging and cancer
Source: PLoS One. 2019 Jan 25;14(1):e0210863. doi: 10.1371/journal.pone.0210863 (PMC6347267; doi:10.1371/journal.pone.0210863)
Supplement: S2 Table — Lifelong aerobic exercise training (AET) mitigates age-associated muscle loss and testicular atrophy in C57BL/J6 mice. Age-associated organ wasting was isolated to fast-twitch muscles and testicles (test.), while slow-twitch muscles (solues; sol.) and other internal organs (heart, brain, liver, kidney, spleen, and lungs) were largely spared or increased in mass. Major thigh (quadriceps complex) and lower leg (anterior and posterior crural) muscles were summed to obtain total hindlimb muscle (HL) mass. *Significant effects of aging, †lifelong aerobic exercise training, and ‡gender (P ≤ 0.05). (DOC) [file pone.0210863.s004.doc]

| **Group** | **BW (g)**  ***†‡** | **Soleus  (mg)**  **†‡** | **Soleus/BW**  **(mg/g)**  ***†** | **EDL  (mg)**  ***‡** | **EDL/BW (mg/g)**  ***** | **Quadriceps  (mg)**  ***‡** | **Quad./BW**  **(mg/g)**  ***†‡** | **Hindlimb (mg)**  ***‡** | **HL/BW**  **(mg/g)**  ***†‡** | **Testicles  (mg)**  ***†** | **Test./BW**  **(mg/g)**  ***†** |
| --- | --- | --- | --- | --- | --- | --- | --- | --- | --- | --- | --- |
| ***Y-CON***  *(N = 40; 2-mo-old)* |  |  |  |  |  |  |  |  |  |  |  |
| M (20) | 27.3 ± 0.3 | 17.2 ± 0.5 | 0.63 ± 0.02 | 23.1 ± 1.7 | 0.85 ± 0.06 | 425.8 ± 17.4 | 15.6 ± 0.6 | 908.3 ± 24.3 | 33.3 ± 0.7 | 195.5 ± 7.4 | 7.2 ± 0.3 |
| F (20) | 21.3 ± 0.4 | 13.2 ± 1.0 | 0.64 ± 0.05 | 16.9 ± 0.7 | 0.82 ± 0.04 | 291.6 ± 8.2 | 14.2 ± 0.5 | 640.1 ± 19.3 | 31.0 ± 0.7 | NA | NA |
| ***O-SED***  *(N = 32; 26-mo-old)* |  |  |  |  |  |  |  |  |  |  |  |
| M (18) | 36.2 ± 1.1 | 18.4 ± 1.1 | 0.51 ± 0.03 | 18.6 ± 1.4 | 0.51 ± 0.04 | 338.3 ± 9.3 | 9.4 ± 0.5 | 803.5 ± 13.3 | 22.2 ± 1.1 | 167.0 ± 4.3 | 4.7 ± 0.3 |
| F (14) | 31.6 ± 1.3 | 15.3 ± 1.6 | 0.51 ± 0.06 | 14.3 ± 1.9 | 0.47 ± 0.07 | 241.0 ± 12.4 | 8.0 ± 0.5 | 609.4 ± 20.4 | 20.2 ± 1.0 | NA | NA |
| ***O-AET***  *(N = 38; 26-mo-old)* |  |  |  |  |  |  |  |  |  |  |  |
| M (18) | 34.5 ± 0.8 | 20.8 ± 1.7 | 0.64 ± 0.06 | 19.9 ± 2.2 | 0.62 ± 0.08 | 364.1 ± 13.7 | 11.2 ± 0.4 | 848.0 ± 22.2 | 26.1 ± 1.0 | 184.0 ± 7.2 | 5.8 ± 0.3 |
| F (20) | 28.8 ± 0.7 | 17.2 ± 0.6 | 0.60 ± 0.01 | 14.0 ± 1.7 | 0.49 ± 0.08 | 255.2 ± 14.8 | 8.9 ± 0.5 | 617.0 ± 22.9 | 21.6 ± 0.7 | NA | NA |
|  | | | | | | | | | | | |
