# Supplementary material for: Lifelong aerobic exercise protects against inflammaging and cancer
Source: PLoS One. 2019 Jan 25;14(1):e0210863. doi: 10.1371/journal.pone.0210863 (PMC6347267; doi:10.1371/journal.pone.0210863)
Supplement: S3 Table — Lifelong aerobic exercise training (AET) dampens inflammaging in old C57BL/J6 mice. Serum cytokine and chemokine concentrations are reported in pg/mL and expressed as group means ± SE. Each sample consisted of serum from one or two mice within the same experimental condition. All samples were run in duplicate. (DOC) [file pone.0210863.s005.doc]

| **Group (n)** | **GM-CSF** | **IFN-γ** | **IL-1a** | **IL-1b** | **IL-2** | **IL-4** | **IL-5** | **IL-6** | **IL-7** | **IL-10** | **IL-12 (p70)** | **IL-13** | **LIX** | **IL-17a** | **KC** | **MCP-1** | **MIP-2** | **TNF-α** |
| --- | --- | --- | --- | --- | --- | --- | --- | --- | --- | --- | --- | --- | --- | --- | --- | --- | --- | --- |
| ***Y-CON*** |  |  |  |  |  |  |  |  |  |  |  |  |  |  |  |  |  |  |
| M (4) | 20.9  ± 2.0 | 0.47 ± 0.20 | 1887  ± 173 | 7.64 ± 3.89 | 50.09 ± 16.19 | 1.28 ± 0.69 | 22.67 ± 0.46 | 10.66 ± 0.26 | 29.44 ± 3.83 | 7.97 ± 3.90 | 10.63 ± 5.47 | 16.05 ± 1.37 | 32291 ± 1329 | 10.67 ± 0.44 | 231.0 ± 5.0 | 59.28 ± 5.32 | 220.3 ± 5.9 | 12.51 ± 0.91 |
| F (3) | 55.82 ± 20.42 | 5.10 ± 4.98 | 1253 ± 560 | 39.35 ± 3.79 | 120.3 ± 22.9 | 0.78 ± 0.58 | 31.10 ± 2.21 | 53.34 ± 10.01 | 128.8 ± 12.9 | 20.40 ± 2.60 | 35.73 ± 1.99 | 87.62 ± 35.08 | 37658 ± 4349 | 12.47 ± 1.15 | 133.2 ± 45.1 | 99.73 ± 31.24 | 290.6 ± 48.1 | 17.53 ± 5.17 |
| ***Y-CON-EX*** |  |  |  |  |  |  |  |  |  |  |  |  |  |  |  |  |  |  |
| M (4) | 3.33 ± 1.09 | 1.02 ± 0.52 | 1731 ± 156 | 4.61 ± 2.13 | 29.39 ± 11.75 | 1.07 ± 0.42 | 8.88 ± 1.40 | 11.47 ± 3.50 | 22.91 ± 6.03 | 5.18 ± 2.29 | 0.69 ± 0.00 | 13.67 ± 0.00 | 37740 ± 5474 | 5.56 ± 0.35 | 231.2 ± 1.2 | 73.48 ± 10.63 | 278.1 ± 36.5 | 6.53 ± 2.28 |
| F (4) | 23.96 ± 6.38 | 1.94 ± 1.05 | 1635 ± 469 | 18.50 ± 7.35 | 113.8 ± 48.6 | 0.79 ± 0.03 | 23.33 ± 5.44 | 28.53 ± 7.27 | 56.25 ± 18.86 | 14.42 ± 2.22 | 4.46 ± 2.18 | 39.48 ± 14.90 | 42842 ± 64 | 8.67 ± 0.71 | 274.5 ± 22.0 | 44.01 ± 9.86 | 205.9 ± 4.8 | 3.67 ± 2.06 |
| ***O-SED*** |  |  |  |  |  |  |  |  |  |  |  |  |  |  |  |  |  |  |
| M (4) | 95.25 ± 40.64 | 33.14 ± 12.69 | 1689 ± 9 | 13.34 ± 4.37 | 81.43 ± 8.57 | 1.83 ± 0.96 | 12.17 ± 4.39 | 52.61 ± 7.69 | 131.5 ± 48.2 | 27.47 ± 13.31 | 56.66 ± 22.67 | 494.7 ± 275.0 | 42984 ± 5452 | 15.27 ± 0.69 | 356.5 ± 49.7 | 296.1 ± 4.1 | 286.7 ± 28.8 | 24.33 ± 3.42 |
| F (4) | 51.50 ± 10.46 | 0.84 ± 0.42 | 2366 ± 564 | 96.75 ± 55.33 | 112.0 ± 14.6 | 3.89 ± 1.39 | 52.42 ± 11.02 | 29.71 ± 17.02 | 44.59 ± 6.35 | 30.30 ± 17.31 | 29.43 ± 1.18 | 145.3 ± 76.0 | 35375 ± 5913 | 19.67 ± 5.15 | 234.3 ± 34.5 | 237.7 ± 30.4 | 286.8 ± 40.4 | 42.56 ± 12.24 |
| ***O-SED-EX*** |  |  |  |  |  |  |  |  |  |  |  |  |  |  |  |  |  |  |
| M (2) | 184.6 ± 0.00 | 17.52 ± 0.00 | 4219 ± 0.00 | 145.2 ± 0.00 | 198.8 ± 0.00 | 12.02 ± 0.00 | 65.35 ± 0.00 | 188.9 ± 0.00 | 262.7 ± 0.00 | 162.1 ± 0.00 | 254.5 ± 0.00 | 633.9 ± 0.00 | 40941 ± 0.00 | 26.95 ± 0.00 | 524.7 ± 0.00 | 202.4 ± 0.00 | 566.6 ± 0.00 | 47.50 ± 0.00 |
| F (4) | 65.44 ± 2.03 | 0.12 ± 0.00 | 2321 ± 51 | 76.21 ± 11.55 | 170.3 ± 33.8 | 23.03 ± 12.01 | 93.42 ± 42.66 | 240.2 ± 112.8 | 67.71 ± 39.03 | 303.8 ± 150.3 | 253.2 ± 120.3 | 213.7 ± 76.1 | 38785 ± 2659 | 33.03 ± 13.05 | 289.04 ± 82.46 | 151.6 ± 1.3 | 240.9 ± 2.5 | 30.26 ± 2.69 |
| ***O-AET*** |  |  |  |  |  |  |  |  |  |  |  |  |  |  |  |  |  |  |
| M (4) | 31.70 ± 0.50 | 0.12 ± 0.00 | 2121 ± 74 | 14.15 ± 7.64 | 68.99 ± 13.26 | 0.68 ± 0.16 | 9.52 ± 0.08 | 30.82 ± 10.53 | 63.57 ± 8.98 | 14.95 ± 7.85 | 0.69 ± 0.00 | 13.67 ± 0.00 | 71885 ± 3309 | 12.21 ± 2.33 | 445.4 ± 8.1 | 117.0 ± 4.4 | 254.9 ± 13.0 | 11.97 ± 5.32 |
| F (5) | 40.18 ± 13.48 | 3.85 ± 2.29 | 1725 ± 27 | 43.80 ± 13.93 | 62.21 ± 23.39 | 3.02 ± 0.96 | 32.00 ± 3.73 | 37.53 ± 15.27 | 133.0 ± 26.7 | 56.32 ± 15.87 | 47.95 ± 17.38 | 77.95 ± 39.36 | 24859 ± 10069 | 9.97 ± 0.25 | 301.3 ± 21.9 | 187.9 ± 52.1 | 280.3 ± 37.7 | 24.96 ± 5.10 |
| ***O-AET-EX*** |  |  |  |  |  |  |  |  |  |  |  |  |  |  |  |  |  |  |
| M (3) | 77.87 ± 38.22 | 14.78 ± 7.33 | 2838 ± 1070 | 94.18 ± 30.38 | 142.6 ± 27.7 | 6.88 ± 2.64 | 56.23 ± 3.25 | 89.48 ± 44.63 | 244.2 ± 6.5 | 57.06 ± 20.39 | 120.4 ± 59.8 | 173.4 ± 66.2 | 47671 ± 6579 | 22.47 ± 6.23 | 586.7 ± 60.9 | 180.8 ± 28.7 | 508.1 ± 158.0 | 36.75 ± 7.69 |
| F (5) | 31.21 ± 4.48 | 0.12 ± 0.00 | 1937 ± 37 | 49.22 ± 15.75 | 76.81 ± 7.56 | 3.89 ± 2.55 | 55.17 ± 24.78 | 35.08 ± 25.32 | 52.79 ± 14.28 | 39.19 ± 9.81 | 32.64 ± 27.66 | 59.19 ± 29.87 | 36394 ± 5191 | 11.22 ± 0.69 | 313.1 ± 70.8 | 78.60 ± 13.60 | 207.3 ± 11.3 | 23.83 ± 2.06 |
