# Supplementary material for: Lifelong aerobic exercise protects against inflammaging and cancer
Source: PLoS One. 2019 Jan 25;14(1):e0210863. doi: 10.1371/journal.pone.0210863 (PMC6347267; doi:10.1371/journal.pone.0210863)
Supplement: S4 Table — (DOC) [file pone.0210863.s006.doc]

| **ANOVA (p-values)** | **GM-CSF** | **IFN-γ** | **IL-1a** | **IL-1b** | **IL-2** | **IL-4** | **IL-5** | **IL-6** | **IL-7** | **IL-10** | **IL-12 (p70)** | **IL-13** | **LIX** | **IL-17a** | **KC** | **MCP-1** | **MIP-2** | **TNF-α** |
| --- | --- | --- | --- | --- | --- | --- | --- | --- | --- | --- | --- | --- | --- | --- | --- | --- | --- | --- |
| **MAIN EFFECTS** |  |  |  |  |  |  |  |  |  |  |  |  |  |  |  |  |  |  |
| *AGE* | ***<0.001*** | ***0.010*** | ***<0.001*** | ***<0.001*** | ***0.004*** | ***0.016*** | ***0.015*** | ***0.006*** | ***0.002*** | ***0.013*** | ***<0.001*** | ***<0.001*** | *0.545* | ***0.002*** | ***0.001*** | ***0.001*** | ***0.001*** | ***<0.001*** |
| *AET* | ***0.001*** | ***0.041*** | *0.067* | *0.084* | ***0.002*** | *0.069* | *0.253* | ***0.027*** | *0.873* | ***0.044*** | ***0.014*** | ***0.002*** | *0.250* | ***0.024*** | *0.140* | ***0.001*** | *0.373* | ***0.015*** |
| *EX* | *0.311* | *0.229* | ***0.013*** | *0.201* | *0.068* | ***0.050*** | *0.174* | ***0.024*** | *0.336* | ***0.033*** | ***0.014*** | *0.651* | *0.339* | *0.333* | ***0.009*** | ***0.001*** | ***0.019*** | *0.582* |
| *GENDER* | *0.051* | ***0.009*** | *0.065* | *0.376* | *0.055* | *0.392* | *0.092* | *0.513* | *0.064* | *0.354* | *1.000* | *0.063* | *0.954* | *0.355* | ***0.004*** | ***0.050*** | ***0.001*** | *0.846* |
| **INTERACTIONS** |  |  |  |  |  |  |  |  |  |  |  |  |  |  |  |  |  |  |
| *AGE*EX* | ***0.008*** | *0.379* | ***0.035*** | *0.054* | ***0.016*** | ***0.048*** | ***0.036*** | ***0.011*** | ***0.006*** | ***0.027*** | ***0.004*** | *0.460* | *0.459* | ***0.049*** | *0.526* | ***0.008*** | ***0.004*** | *0.069* |
| *AET*EX* | *0.262* | *0.085* | *0.143* | *0.723* | *0.153* | *0.120* | *0.673* | ***0.042*** | *0.508* | ***0.031*** | ***0.042*** | *0.842* | *0.473* | *0.410* | *0.663* | *0.129* | *0.711* | *0.484* |
| *AGE*GENDER* | ***<0.001*** | ***0.002*** | *0.628* | *0.643* | *0.061* | *0.336* | *0.388* | *0.816* | ***<0.001*** | *0.491* | *0.689* | ***0.019*** | *0.114* | *0.735* | ***0.026*** | ***0.019*** | ***<0.001*** | *0.942* |
| *AET*GENDER* | ***0.040*** | ***0.017*** | *0.942* | *0.684* | *0.219* | *0.330* | *0.446* | *0.578* | *0.058* | *0.476* | *0.936* | ***0.040*** | ***0.019*** | *0.151* | *0.709* | *0.369* | *0.730* | *0.959* |
| *EX*GENDER* | *0.099* | *0.472* | *0.055* | ***0.018*** | *0.567* | *0.520* | *0.905* | *0.717* | ***0.033*** | *0.448* | *0.975* | *0.737* | *0.677* | *0.856* | *0.829* | *0.201* | ***<0.001*** | ***0.013*** |
| *AGE*GENDER*EX* | *0.253* | *0.237* | ***0.005*** | *0.055* | *0.353* | *0.541* | *0.728* | *0.460* | *0.587* | *0.427* | *0.742* | *0.942* | *0.646* | *0.982* | *0.057* | *0.115* | ***0.036*** | *0.099* |
| *AET*GENDER*EX* | *0.727* | ***0.038*** | *0.055* | *0.292* | *1.000* | *0.310* | *0.837* | *0.326* | *0.069* | *0.247* | *0.285* | *0.747* | *0.129* | *0.514* | *0.920* | ***0.045*** | *0.997* | *0.600* |
|  |  |  |  |  |  |  |  |  |  |  |  |  |  |  |  |  |  |  |
