# Supplementary material for: Lifelong aerobic exercise protects against inflammaging and cancer
Source: PLoS One. 2019 Jan 25;14(1):e0210863. doi: 10.1371/journal.pone.0210863 (PMC6347267; doi:10.1371/journal.pone.0210863)
Supplement: S5 Table — Lifelong aerobic exercise training (AET) mitigates serum SPARC levels in old C57BL/J6 mice. Serum myokine concentrations are in pg/mL and expressed as group means ± SE. Each sample consisted of serum from one or two mice within the same experimental condition. All samples were run in duplicate. (DOC) [file pone.0210863.s007.doc]

| **Group (n)** | **Osteonectin**  **(SPARC)** | **Osteocrin**  **(Musclin)** | **Follistatin-like protein 1**  **(FSTL-1)** | **Myostatin**  **(MSTN)** | **FGF-21** | **Fractalkine**  **(CX3CL1)** | **IL-15** |
| --- | --- | --- | --- | --- | --- | --- | --- |
| ***Y-CON*** |  |  |  |  |  |  |  |
| M (4) | 101753 ± 8989 | 77.07 ± 5.91 | 3251 ± 430 | 3021 ± 303 | 253.8 ± 128.7 | 73.04 ± 20.89 | 176.3 ± 46.4 |
| F (3) | 46897 ± 17368 | 83.48 ± 3.19 | 1635 ± 630 | 4000 ± 1981 | 152.5 ± 65.7 | 243.3 ± 160.3 | 959.2 ± 351.2 |
| ***Y-CON-EX*** |  |  |  |  |  |  |  |
| M (4) | 106880 ± 13195 | 61.43 ± 1.86 | 3801 ± 373 | 6405 ± 1879 | 176.6 ± 3.9 | 92.56 ± 6.88 | 122.5 ± 36.7 |
| F (4) | 75987 ± 13066 | 67.87 ± 18.31 | 1688 ± 538 | 1957 ± 391 | 216.0 ± 73.8 | 67.49 ± 2.06 | 310.5 ± 119.1 |
| ***O-SED*** |  |  |  |  |  |  |  |
| M (4) | 102309 ± 2174 | 52.44 ± 9.95 | 702.0 ± 383.5 | 444.6 ± 225.1 | 96.82 ± 11.23 | 67.47 ± 3.49 | 221.4 ± 20.4 |
| F (4) | 201480 ± 77942 | 68.23 ± 1.43 | 305.7 ± 154.7 | 60.34 ± 0.00 | 95.07 ± 24.06 | 14.28 ± 2.46 | 65.3 ± 22.9 |
| ***O-SED-EX*** |  |  |  |  |  |  |  |
| M (2) | 79196 ± 0.0 | 134.2 ± 0.0 | 506.2 ± 0.0 | 2277 ± 0.0 | 180.2 ± 0.0 | 133.3 ± 0.0 | 670.4 ± 0.0 |
| F (4) | 189029 ± 15605 | 82.80 ± 19.71 | 37.71 ± 0.00 | 222.6 ± 93.7 | 313.2 ± 116.3 | 104.6 ± 19.3 | 186.5 ± 101.1 |
| ***O-AET*** |  |  |  |  |  |  |  |
| M (4) | 88375 ± 10951 | 60.81 ± 9.58 | 759.3 ± 416.6 | 688.7 ± 247.6 | 22.48 ± 12.33 | 75.88 ± 2.75 | 89.8 ± 45.3 |
| F (5) | 79063 ± 9586 | 52.57 ± 16.31 | 490.4 ± 277.2 | 163.3 ± 56.9 | 23.29 ± 1.18 | 28.78 ± 9.53 | 17.0 ± 3.5 |
| ***O-AET-EX*** |  |  |  |  |  |  |  |
| M (3) | 86877 ± 18028 | 85.17 ± 3.17 | 46.34 ± 4.32 | 582.2 ± 88.4 | 109.603 ± 0.57 | 99.64 ± 28.78 | 221.6 ± 17.7 |
| F (5) | 80399 ± 4762 | 145.9 ± 15.7 | 499.83 ± 115.59 | 675.3 ± 95.02 | 86.811 ± 16.95 | 67.94 ± 16.16 | 202.6 ± 93.2 |
