# Supplementary material for: Lifelong aerobic exercise protects against inflammaging and cancer
Source: PLoS One. 2019 Jan 25;14(1):e0210863. doi: 10.1371/journal.pone.0210863 (PMC6347267; doi:10.1371/journal.pone.0210863)
Supplement: S6 Table — (DOC) [file pone.0210863.s008.doc]

| **ANOVA (p-values)** | **Osteonectin**  **(SPARC)** | **Osteocrin**  **(Musclin)** | **Follistatin-like protein 1 (FSTL-1)** | **Myostatin**  **(MSTN)** | **FGF-21** | **Fractalkine**  **(CX3CL1)** | **IL-15** |
| --- | --- | --- | --- | --- | --- | --- | --- |
| ***MAIN EFFECTS*** |  |  |  |  |  |  |  |
| *AGE* | ***0.020*** | *0.175* | ***<0.001*** | ***<0.001*** | *0.621* | *0.259* | *0.234* |
| *AET* | ***0.014*** | *0.870* | *0.751* | ***0.040*** | ***0.003*** | *0.253* | ***0.002*** |
| *EX* | *0.989* | *0.070* | *0.904* | *0.243* | *0.217* | *1.000* | *0.707* |
| *GENDER* | *0.213* | *0.512* | ***<0.001*** | ***0.046*** | *0.762* | *0.644* | *0.352* |
| ***INTERACTIONS*** |  |  |  |  |  |  |  |
| AGE*EX | *0.475* | ***0.001*** | *0.362* | *0.816* | *0.178* | ***0.031*** | ***0.001*** |
| *AET*EX* | *0.694* | *0.607* | *0.756* | ***<0.001*** | *0.263* | ***0.030*** | *0.174* |
| AGE*GENDER | ***0.006*** | *0.169* | ***0.021*** | *0.715* | *0.403* | *0.108* | ***<0.001*** |
| AET*GENDER | ***0.019*** | ***0.042*** | *0.180* | ***<0.001*** | *0.256* | *0.940* | ***0.006*** |
| *EX*GENDER* | *0.722* | *0.062* | *0.624* | ***0.019*** | *0.237* | *0.221* | ***0.015*** |
| *AGE*GENDER*EX* | *0.891* | *0.062* | *0.714* | *0.191* | *0.979* | *0.119* | *0.450* |
| *AET*GENDER*EX* | *0.931* | ***0.003*** | *0.306* | ***<0.001*** | *0.241* | *0.823* | ***0.045*** |
|  |  |  |  |  |  |  |  |
