# Supplementary material for: Lifelong aerobic exercise protects against inflammaging and cancer
Source: PLoS One. 2019 Jan 25;14(1):e0210863. doi: 10.1371/journal.pone.0210863 (PMC6347267; doi:10.1371/journal.pone.0210863)
Supplement: S8 Table — (DOCX) [file pone.0210863.s010.docx]

**RNA isolation and reverse transcription**

RNA was isolated from 15–25 mg of muscle tissue and/or heart tissue using the Trizol/RNeasy method. All samples were homogenized with 1 mL of Trizol Reagent (Life Technologies, Burlington, ON, Canada), in Lysing Maxtrix D tubes (MP Biomedicals, Solon, OH, USA), with the FastPrep-24 Tissue and Cell Homogenizer (MP Biomedicals) for a duration of 40 s at a setting of 6 m s^−1^. Following a 5 min room temperature incubation, homogenized samples were stored at −80°C for 1 month until further processing. After thawing on ice, 200 mL of chloroform (Sigma-Aldrich) was added to each sample, mixed vigorously for 15 s, incubated at room temperature for 5 min, and spun at 12,000 *g* for 10 min at 4°C. The RNA (aqueous) phase was purified using the EZNA Total RNA Kit 1 (Omega Bio-Tek, Norcross, GA, USA) as per the manufacturer's instructions. RNA concentration (ng mL^−1^) and purity (260/280) was determined with the Nano‐Drop 1000 Spectrophotometer (Thermo Fisher Scientific, Waltham, MA, USA). Samples were then reverse transcribed using a high capacity cDNA reverse transcription kit (SuperScript® VILO™ Master Mix; Invitrogen, cat. no. 11755050).

**Quantitative PCR**

The levels of expression of genes were assayed using TaqMan® Fast Advanced Master Mix (cat. no. 4444963) and TaqMan Gene Expression Assays (*S8 Table)* performed in a 7900HT Fast Real-Time PCR System (Bio-RAD, USA). Messenger RNA expression was calculated using the 2^-ΔCT^  method (Livak & Schmittgen, [2008](https://physoc.onlinelibrary.wiley.com/doi/full/10.1113/JP275155#tjp12784-bib-0024)). Briefly, *C*_t_ values were first normalized to the housekeeping gene glyceraldehyde-3-phosphate dehydrogenase (GAPDH) (*S8 Table*). *C*_t_ values normalized to GAPDH were expressed as Δ*C*_t_ values. Values were then transformed out of the logarithmic scale using the formula 2^-ΔCT^ (Livak & Schmittgen, [2008](https://physoc.onlinelibrary.wiley.com/doi/full/10.1113/JP275155#tjp12784-bib-0024)). Thus, mRNA values are expressed 2^-ΔCT^ (mean ± SEM).

| **Gene** | **Thermo Scientific Catalogue #** |
| --- | --- |
| NLRP3 | Mm00840904_m1 |
| CDKN2A | Mm00494449_m1 |
| IL-1β | Mm00434228_m1 |
| IL-18 | Mm00434226_m1 |
| IL-6 | Mm00446190_m1 |
| TNFα | Mm00443258_m1 |
| GAPDH | Mm99999915_g1 |

# References

# [Schmittgen TD](https://www.ncbi.nlm.nih.gov/pubmed/?term=Schmittgen%20TD%5BAuthor%5D&cauthor=true&cauthor_uid=18546601), [Livak KJ](https://www.ncbi.nlm.nih.gov/pubmed/?term=Livak%20KJ%5BAuthor%5D&cauthor=true&cauthor_uid=18546601). Analyzing real-time PCR data by the comparative C(T) method. [Nat Protoc.](https://www.ncbi.nlm.nih.gov/pubmed/18546601)2008;3(6):1101-8.

.
