# Supplementary material for: Lifelong aerobic exercise protects against inflammaging and cancer
Source: PLoS One. 2019 Jan 25;14(1):e0210863. doi: 10.1371/journal.pone.0210863 (PMC6347267; doi:10.1371/journal.pone.0210863)
Supplement: Supplementary file 9 [file pone.0210863.s001.doc]

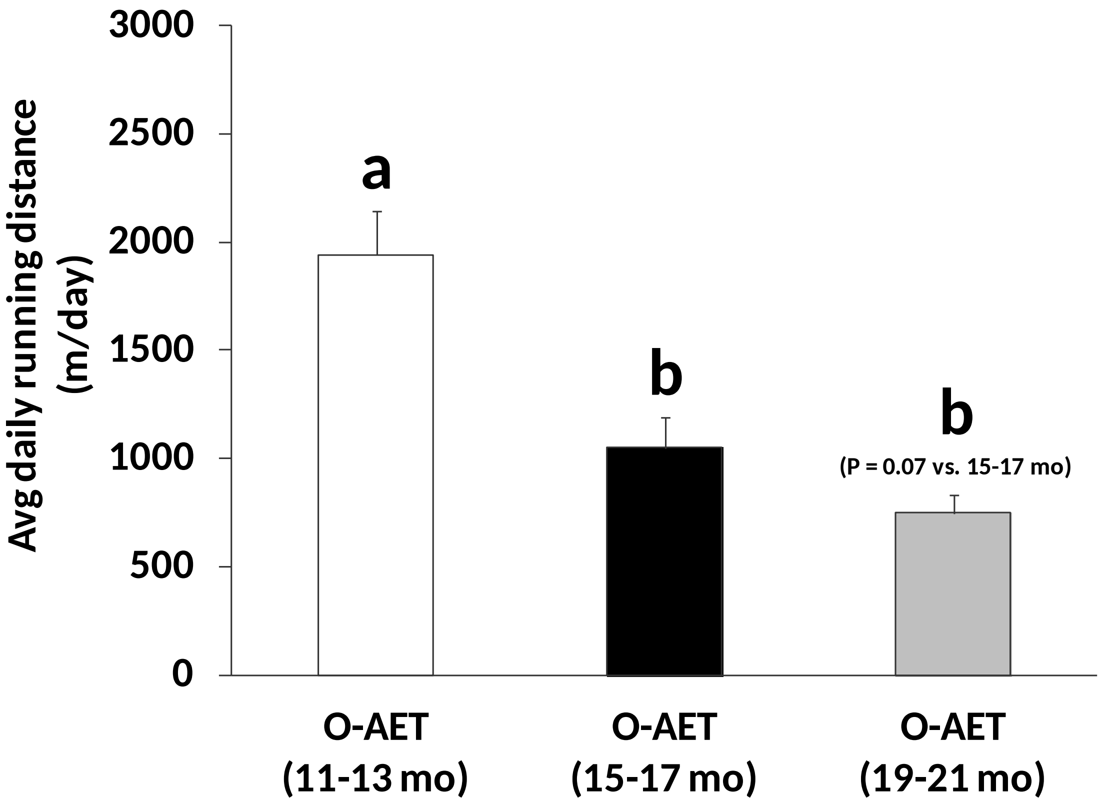

Avg daily running distance (m/day)
3000
2500
2000
1500
1000
500
0
a
b
b
(P = 0.07 vs. 15-17 mo)
O-AET (11-13 mo)
O-AET (15-17 mo)
O-AET (19-21 mo)
