# Supplementary material for: Lifelong aerobic exercise protects against inflammaging and cancer
Source: PLoS One. 2019 Jan 25;14(1):e0210863. doi: 10.1371/journal.pone.0210863 (PMC6347267; doi:10.1371/journal.pone.0210863)
Supplement: Supplementary file 10 [file pone.0210863.s009.doc]

Group	GM-CSF	IL-1α	IL-2	IL-6	Osteonectin (SPARC)	Osteocrin (Musclin)	Follistatin-like protein 1 (FSTL1)	Myostatin (MSTN)	FGF-21	LIX
Y-CON2 (N = 5)	3.7	981.3	20.9	28.5	62075.4	38.1	1765.0	4651.1	222.0	30035.3
Y-CON-EX2 (N = 5)	23.1	1113.6	29.7	59.1	76511.3	61.4	2720.4	3228.6	285.1	41463.5
**P-value**	5.24E-03	5.27E-03	5.46E-02	4.79E-08	2.35E-05	3.77E-02	1.16E-04	1.71E-03	5.52E-03	1.07E-09
**Exercise effect**	524% ↑	13% ↑	42% ↑	107% ↑	23% ↑	61% ↑	54% ↑	31% ↓	28% ↑	38% ↑
